# Metformin regulates adiponectin signalling in epicardial adipose tissue and reduces atrial fibrillation vulnerability

**DOI:** 10.1111/jcmm.15407

**Published:** 2020-05-22

**Authors:** Biao Li, Sunny S. Po, Baojian Zhang, Fan Bai, Jiayi Li, Fen Qin, Na Liu, Chao Sun, Yichao Xiao, Tao Tu, Shenghua Zhou, Qiming Liu

**Affiliations:** ^1^ Department of Cardiology/Cardiac Catheterization Lab Second Xiangya Hospital Central South University Changsha City Hunan Province China; ^2^ Heart Rhythm Institute and Department of Medicine University of Oklahoma Health Sciences Center Oklahoma City OK USA; ^3^ Department of Cardiology the Affiliated Chinese Medicine Hospital of Xinjiang Medical University Urumqi City Xinjiang Province China

**Keywords:** adiponectin, atrial fibrillation, epicardial adipose tissue, inflammation, metformin

## Abstract

Epicardial adipose tissue (EAT) remodelling is closely related to the pathogenesis of atrial fibrillation (AF). We investigated whether metformin (MET) prevents AF‐dependent EAT remodelling and AF vulnerability in dogs. A canine AF model was developed by 6‐week rapid atrial pacing (RAP), and electrophysiological parameters were measured. Effective refractory periods (ERP) were decreased in the left and right atrial appendages as well as in the left atrium (LA) and right atrium (RA). MET attenuated the RAP‐induced increase in ERP dispersion, cumulative window of vulnerability, AF inducibility and AF duration. RAP increased reactive oxygen species (ROS) production and nuclear factor kappa‐B (NF‐κB) phosphorylation; up‐regulated interleukin‐6 (IL‐6), tumour necrosis factor‐α (TNF‐α) and transforming growth factor‐β1 (TGF‐β1) levels in LA and EAT; decreased peroxisome proliferator‐activated receptor gamma (PPARγ) and adiponectin (APN) expression in EAT and was accompanied by atrial fibrosis and adipose infiltration. MET reversed these alterations. In vitro*,* lipopolysaccharide (LPS) exposure increased IL‐6, TNF‐α and TGF‐β1 expression and decreased PPARγ/APN expression in 3T3‐L1 adipocytes, which were all reversed after MET administration. Indirect coculture of HL‐1 cells with LPS‐stimulated 3T3‐L1 conditioned medium (CM) significantly increased IL‐6, TNF‐α and TGF‐β1 expression and decreased SERCA2a and p‐PLN expression, while LPS + MET CM and APN treatment alleviated the inflammatory response and sarcoplasmic reticulum Ca^2+^ handling dysfunction. MET attenuated the RAP‐induced increase in AF vulnerability, remodelling of atria and EAT adipokines production profiles. APN may play a key role in the prevention of AF‐dependent EAT remodelling and AF vulnerability by MET.

## 1 INTRODUCTION

Atrial fibrillation (AF), the most common arrhythmia, is associated with increased morbidity and mortality.^1^ Epicardial adipose tissue (EAT) is an active endocrine and paracrine organ which is in direct contact with the atria and shares a common blood supply with the myocardium. The important role of EAT in AF genesis and perpetuation has been investigated.^2^, ^3^ EAT may lead to AF via atrial structural and electrical remodelling via various mechanisms.^4^ EAT infiltration alters the atrial electrophysiological properties, while various adipokines secreted by EAT influence AF pathogenesis. In the disease state, such as AF, the EAT secretome profile is remodelled. This is characterized by a decrease in the release of homeostatic protective factors and an increase in the release of pathological adipokines.^4^, ^5^ These activities promote atrial inflammation and lead to structural and electrical remodelling.^6^, ^7^ Although inflammation is a known contributor to AF, it is difficult to elucidate the anti‐inflammatory effects of AF therapy. EAT, as a transducer of inflammation, may be a strong candidate for anti‐inflammatory therapeutic intervention.^8^

Metformin (MET) exhibits anti‐inflammatory^9^, ^10^, ^11^ and anti‐oxidative stress^12^ properties, which are greatly involved in the complex and multifactorial pathogenesis of AF. MET also influences EAT accumulation,^13^ adipogenesis and adipocyte function, including the production and release of adipokines.^14^, ^15^ MET is associated with a decreased risk of AF in patients with type 2 diabetes.^12^ Whether MET offers the same protection in metabolically normal patients and the underlying mechanisms remain unclear.

We hypothesized that MET decreases the incidence of AF by reversing AF‐dependent EAT remodelling. The aims of this study were as follows: (a) to determine the impact of AF on EAT remodelling and the role of AF‐dependent EAT remodelling in atrial fibrosis and AF maintenance; (b) to evaluate whether MET reverses AF‐dependent EAT remodelling.

## 2 METHODS

### 2.1 Animal preparation

This study was performed in strict accordance with the recommendations in the Guide for the Care and Use of Laboratory Animals of the National Institutes of Health. The protocol was approved by the Committee governing the Ethics of Animal Experiments of the Wuhan University. Dogs were anesthetized with 3% sodium pentobarbital and ventilated with a positive‐pressure respirator (MAO01746; Harvard Apparatus). The initial dose of sodium pentobarbital was 1 mL/kg and an additional 2 ml/h was administered. All efforts were made to minimize suffering.

### 2.2 Group setting

Eighteen male beagle dogs (weight, 8‐10 kg) were supplied by the Center of Experimental Animals at the Medical College of Wuhan University. Animals' age and bodyweight before after interventions are presented in Table S1. Dogs were randomly divided into three groups: (a) sham‐operated (normal diet without rapid atrial pacing (RAP), n = 6), (b) RAP (RAP without MET, n = 6) and (c) RAP + MET (RAP with MET). Consistent with previous reports,^16^ the daily oral administration of MET (100 mg/kg; Squibb Pharmaceutical) was initiated 1 week prior to surgery and continued throughout the study period.

### 2.3 Canine model of atrial fibrillation

AF was induced in canine using long‐term RAP.^17^ In brief, a programmable pacemaker (AOO, Harbin University of Science and Technology) was implanted and used for continuous atria pacing at 400 bpm for 6 weeks to induce AF. The success of this procedure was confirmed by electrocardiography. Sham‐operated dogs were implanted with a same instrument, but without pacemaker activation.

### 2.4 Atrial electrophysiological study

Standard ECG limb leads were recorded at the baseline before pacemaker implantation and after 6‐week RAP. Left and right‐sided thoracotomies were performed at the fourth intercostal space. Multielectrode catheters were secured to allow pacing and recorded from the left and right atrial appendage (LAA and RAA) and left and right atria (LA and RA). The electrophysiological parameters, including effective refractory period (ERP), ERP dispersion, window of vulnerability (WOV) and AF duration, were measured as previously described.^18^ Programmed stimulation of the atrial myocardium was performed using a computer‐based Lab System (Lead 7000; Jinjiang, China). ERP was determined by programmed pacing with 8 consecutive stimuli (S1‐S1 = 300 ms) followed by a premature stimulus (S1‐S2), which was progressively decreased until refractoriness was achieved. Pacing was performed at 2 × threshold (TH). ERP dispersion was calculated offline as the coefficient of variation (standard deviation/mean) of ERP at all recording sites.^19^ The difference between longest and shortest S1‐S2 interval where AF was induced at each bipolar pair was defined as the WOV, which serves as a quantitative measure of AF inducibility.^19^ Cumulative WOV was the sum of WOVs from all sites in each dog. AF was defined as an irregular atrial rate faster than 500 beats/min associated with irregular atrioventricular conduction lasting >5 seconds.^20^ To determine AF vulnerability, 10 consecutive bursts of rapid atrial pacing (cycle length 60 ms) at 4 sites for 2 seconds were implemented at 30 seconds intervals. AF duration induced by burst pacing from all episodes in each dog was analysed. AF inducibility was defined as the rate of first irregular rhythm lasting >5 seconds after cessation of burst pacing. The electrophysiological study was performed by operators blinded to the treatment group.

After 6 weeks, electrophysiological parameters were remeasured, anaesthetized animals were killed and hearts were removed. Left atrial posterior wall and adjacent EAT samples were collected and immersed in 4% paraformaldehyde or snap‐frozen at −80°C for further analysis. Fasting blood samples were collected at the baseline and before measuring electrophysiological parameters and stored at −80°C for further analysis.

### 2.5 Cell culture

Murine atrial myocytes, HL‐1, were provided by Dr Li (Central South University) with the permission of Dr Claycomb (Louisiana State University Health Sciences Center). Cells were cultured with Claycomb Medium supplemented with 10% foetal bovine serum (FBS), 100 U/mL penicillin/streptomycin, 0.1 mmol/L norepinephrine and 2 mmol/L L‐glutamine. Mouse 3T3‐L1 pre‐adipocytes (Amerrican Type Culture Collection −173^TM^) were cultured and maintained in Dulbecco's modified Eagle's medium (Gibco) with 25 mmol/L glucose, supplemented with 10% FBS and 100 U/mL penicillin‐streptomycin. HL‐1 and 3T3‐L1 were incubated in a humidified atmosphere with 5% CO_2_ at 37°C.

To initiate adipocyte differentiation, we used a previously described protocol.^21^ In brief, confluent pre‐adipocytes were differentiated for 48 hours in complete medium supplemented with 1 μmol/L dexamethasone (Sigma), 0.5 mmol/L isobutylmethylxanthine (Sigma), and 10 μg/mL insulin (Procell). The medium was then replaced with complete medium and insulin for 48 hours. Four days after confluence, media were replaced with complete medium and changed every 2 days until day 9, when cells acquired the morphology and typical features of mature adipocytes.

### 2.6 In vitro treatments

To detect the inhibitory effect of MET on adipocytes inflammation, plated 3T3‐L1 mature adipocytes were incubated with or without 4 mmol/L MET, the physiological dose,^22^ for 12 hours and then exposed to 1 µg/mL lipopolysaccharide (LPS; Sigma) for 24 hours to stimulate inflammatory response. In brief, 3T3‐L1 mature adipocytes were exposed to four conditions: negative control medium, LPS at 1 µg/mL, MET at 4 mmol/L + LPS at 1 µg/mL, and MET at 4 mmol/L. All experiments were conducted in the absence of FBS and antibiotics.

To detect the effect of MET on the interactions between HL‐1 cells and 3T3‐L1 mature adipocytes, HL‐1 cells were indirectly co‐cultured with 3T3‐L1 via an exchange medium. As previously described, 3T3‐L1 mature adipocytes were incubated with 1 µg/mL LPS for 24 hours with or without pre‐treatment with 4 mmol/L MET for 12 hours. The adipocytes were then washed with phosphate‐buffered saline (PBS) and cultured in fresh FBS‐free medium for another 24 hours. The conditioned medium (CM) of 3T3L1 (Control CM, LPS CM, LPS + MET CM, MET CM) was collected from the four groups after centrifugation (300 rcf, 5 mins). When the plated HL‐1 confluence was 70‐80%, 200 µL of each CM was added to the HL‐1 medium for 48 hours.

LPS and MET treatment affected the concentration of adiponectin (APN) in 3T3‐L1 CM. To validate the role of APN in modulating the interaction between HL‐1 and 3T3‐L1, APN (1 µg/ml) (Sino Biological) was added to the HL‐1 medium when treated with 3T3L1 LPS CM for 48 hours. A schematic diagram of the in vitro experiments is shown in Figure S1.

After the indicated experiments, the supernatants of intracellular proteins were collected with their appropriate reagent and stored at − 80°C for subsequent assays.

### 2.7 Conditioned medium neutralization and immunoprecipitation

3T3‐L1 LPS + MET conditioned medium was neutralized by an adiponectin antibody (Abcam, ab3455). Initially, 2 μg of APN antibody was added to 1 mL of CM and agitated at 35‐50 rpm at 37°C for 1 hour. Then, a 20 μL of resuspended Protein A/G PLUS‐Agarose (sc‐2003; Santa Cruz Biotechnology) was added to the CM. Next, the mixture was incubated and rotated at 4°C overnight. The supernatant (the neutralized CM) was collected by centrifugation (300 rcf, 5 minutes) and used to treat HL‐1 cells.

To assess the potency of the antibody neutralization, the beads were washed with PBS and collected by centrifugation. After washing, the samples were dissolved in a 5 × sodium dodecylsulphate sample buffer by boiling. The immunoprecipitate and supernatants were then separated by sodium dodecyl sulphate‐polyacrylamide gel electrophoresis and immunoblotted using APN antibody.

### 2.8 Histological study

LA and EAT samples were fixed in 4% paraformaldehyde, embedded in paraffin and sliced into 5‑μm‐thick sections. Adipocyte size was examined by calculating its Feret's diameter from haematoxylin and eosin (H&E)‐stained sections.^23^ The extent of interstitial fibrosis of the LA was estimated from Masson‐stained sections and expressed as the percentage of the total area occupied by blue‐stained interstitial tissue. For each section, 5 optical fields were examined using Image‐Pro 6.2 software, and the average value was calculated. Referring to previous reports,^24^ adipose infiltration of the atrium by the overlying EAT was graded on a scale of 0 to 3 based on severity: 0, no infiltration; 1, infiltration to outer third of atrial muscle layer; 2, infiltration up to middle third of atrial wall; and 3, infiltration up to middle or inner third of atrial wall.

### 2.9 Reactive oxygen species (ROS)detection

ROS content in the LA and EAT tissue was detected by staining the frozen slices of fresh tissue with dihydroethidium (DHE; Beyotime, Shanghai, China) following the manufacturer's instructions. In brief, all sections were stained by DHE (2 µmol/L, 45 minutes, 37°C) in the dark, briefly washed and analysed with a fluorescence microscope. The generation of red fluorescence was used to measure ROS content. For each section, DHE fluorescence was quantified by the mean fluorescence intensity of 5 optical fields using Image‐Pro 6.2 software.

### 2.10 Enzyme immunoassay for cytokines

Quantitative detection of interleukin‐6 (IL‐6), tumour necrosis factor‐α (TNF‐α), transforming growth factor‐β1 (TGF‐β1), adiponectin (APN) plasma concentration, LA myocardium and EAT were performed using a canine‐specific enzyme‐linked immunosorbent assay (ELISA) kit following the manufacturer's protocol (Youersheng, Wuhan, China). In brief, 100 mg of tissue from the LA or EAT was minced and homogenized in 1 mL RIPA lysis buffer (Beyotime). The resulting suspension was sonicated with an ultrasonic cell disrupter to obtain a clear solution. The homogenates were centrifuged for 5 minutes at 10 000 ×g. Supernatants were collected and either assayed immediately or aliquoted and stored at ≤ −20°C.

HL‐1 and 3T3‐L1 cell medium supernatants were collected after centrifugation. APN and TNF‐α concentrations were detected using mouse‐specific ELISA kits according to the manufacturer's protocol.

### 2.11 Cytosolic Ca^2+^ measurement

To detect the effect of 3T3 L1 CM on HL‐1 cytosolic Ca^2+^ homeostasis, cytosolic Ca^2+^ was measured with Fluo‐4 AM (Beyotime, Shanghai, China) according to the manufacturer's instructions. In brief, HL‐1 cells in a 6‐well plate were treated with 3T3L1 CM for 48 hours and then washed with PBS and incubated with 1 μmol/L Fluo‐4 AM for 30 minutes in PBS at room temperature (24°C). They were washed three times with PBS and incubated for an additional 15 minutes in the absence of Fluo‐4AM. The fluorescence intensity was determined at an excitation wavelength of 485 nm and an emission wavelength of 525 nm with a fluorescence microplate reader (Biotek Synergy H4). Representative pictures were taken with a fluorescence microscope.

### 2.12 Biochemical detection

Free fatty acid (FFA) and triglyceride (TG) levels in EAT were measured by biochemical kits (Jiancheng) with the kit instructions.

### 2.13 Western blot analyses and real‐time quantitative PCR

Western blot and qPCR were conducted following standard procedures as described in the Online Supplement. The primer sequences used for real‐time PCR are listed in Table S2.

### 2.14 Statistical analysis

Statistical analyses were performed using R‐3.4.3 (https://www.r‐project.org/). All values are expressed as mean ± SD. Statistically significant differences between means were assessed by ANOVA and Tukey's honestly significant difference test for comparisons between two groups. *P* < .05 was considered statistically significant.

## 3 RESULTS

### 3.1 Electrophysiological testing and AF induction

**Figure 1:**
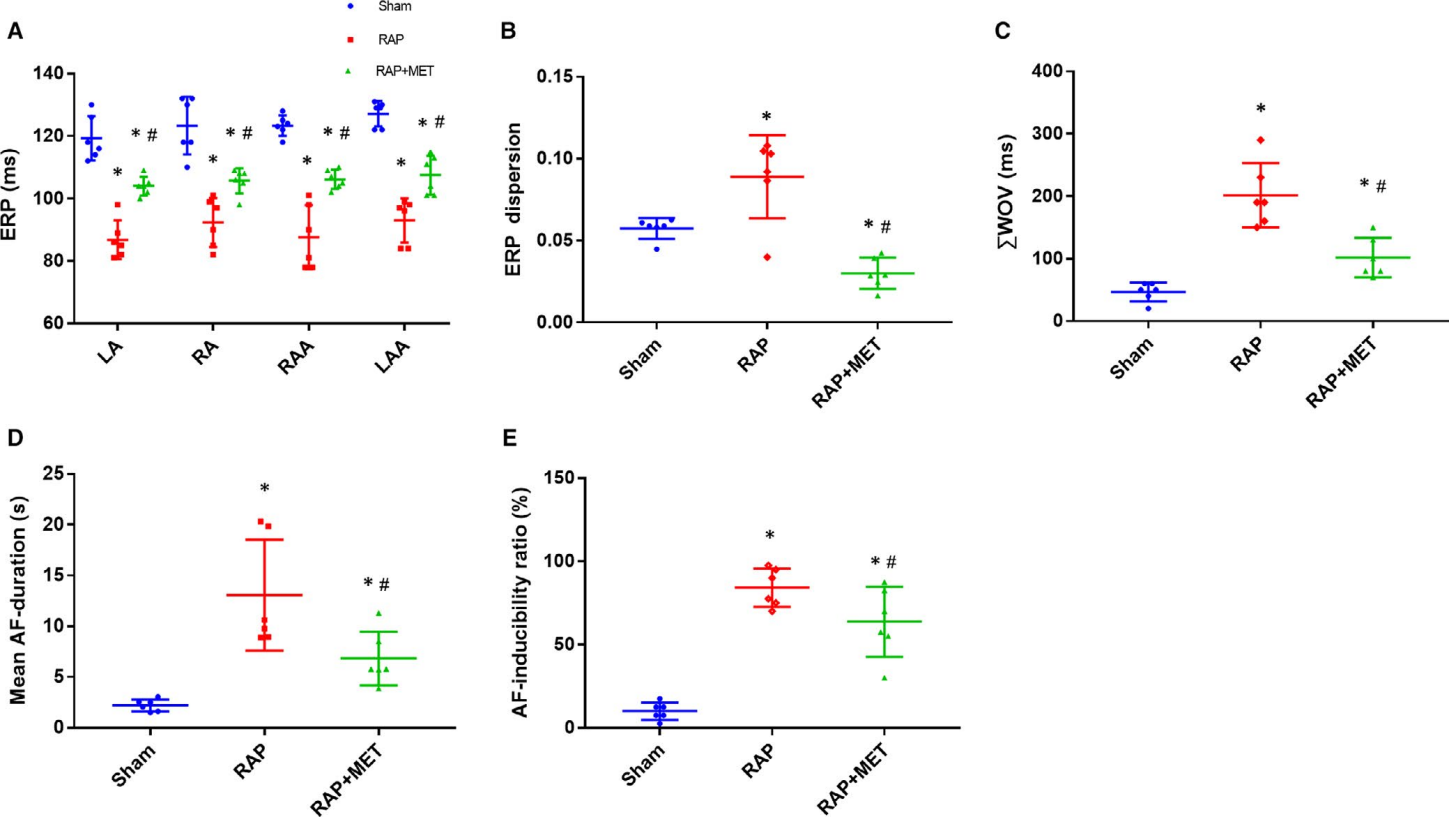
Metformin attenuated RAP‐induced atrial remodelling. A, Changes in mean ERPs in the left atrium (LA), right atrium (RA), left atrial appendage (LAA) and right atrial appendage (RAA). B, Changes in ERP dispersion, (C) cumulative window of vulnerability, (D) mean AF duration and (E) AF inducibility ratio in the sham‐operated group, RAP group, and RAP + MET group (n = 6 animals/group). ^*^ *P* < .05 compared with the sham‐operated group; ^#^ *P* < .05 compared with the RAP group. ERP = effective refractory period; ∑WOV = cumulative window of vulnerability

ERPs at the LA, RA, LAA and RAA are shown in Figure 1A. Except in the sham‐operated group, ERPs at all recording sites significantly decreased after 6 ‐week RAP and MET attenuated the decrease in ERPs. The mean ERPs at the LA site in the sham‐operated, RAP and RAP + MET groups were 119.33 ± 7.11, 86.83 ± 6.17 and 104.00 ± 3.03 ms, respectively (*P* < .05).

ERP dispersion in the RAP group was significantly higher than that in the sham‐operated group (0.09 ± 0.03 vs 0.06 ± 0.01, *P* = .01); MET, however, reversed the increased dispersion (0.09 ± 0.03 to 0.03 ± 0.01, *P* < .01) (Figure 1B).

Quantitative analysis revealed that 6‐week RAP resulted in an increased AF duration (mean AF duration at all recording sites) following burst pacing compared to that in the sham‐operated group (13.06 ± 5.40 vs 2.22 ± 0.59 s, *P* < .01). MET significantly attenuated the increase in AF duration (13.06 ± 5.4 s to 6.82 ± 2.64s, *P* < .01); however, AF duration in the RAP + MET group remained higher than that in the sham‐operated group (*P* = .03) (Figure 1D). AF inducibility in the RAP group was significantly higher than that in the sham‐operated group (84.16% vs 10.00%, *P* < .01); However, MET reduced AF inducibility from 84.16% to 63.75% (*P* = .02) (Figure 1E). In addition, after 6‐week RAP, there was a significant increase in ∑WOV (sum of WOVs at each recording site) compared to sham‐operated group (201.7 ± 51.5 vs 46.7 ± 15.1 ms, *P* < .01), which was attenuated by MET WOV (201.7 ± 51.5 to 101.7 ± 31.9 ms, *P* < .001) (Figure 1C). These data imply that MET could attenuate RAP‐induced atrial electric remodelling.

### 3.2 Effect of metformin on atrial and EAT structure remodelling

**Figure 2:**
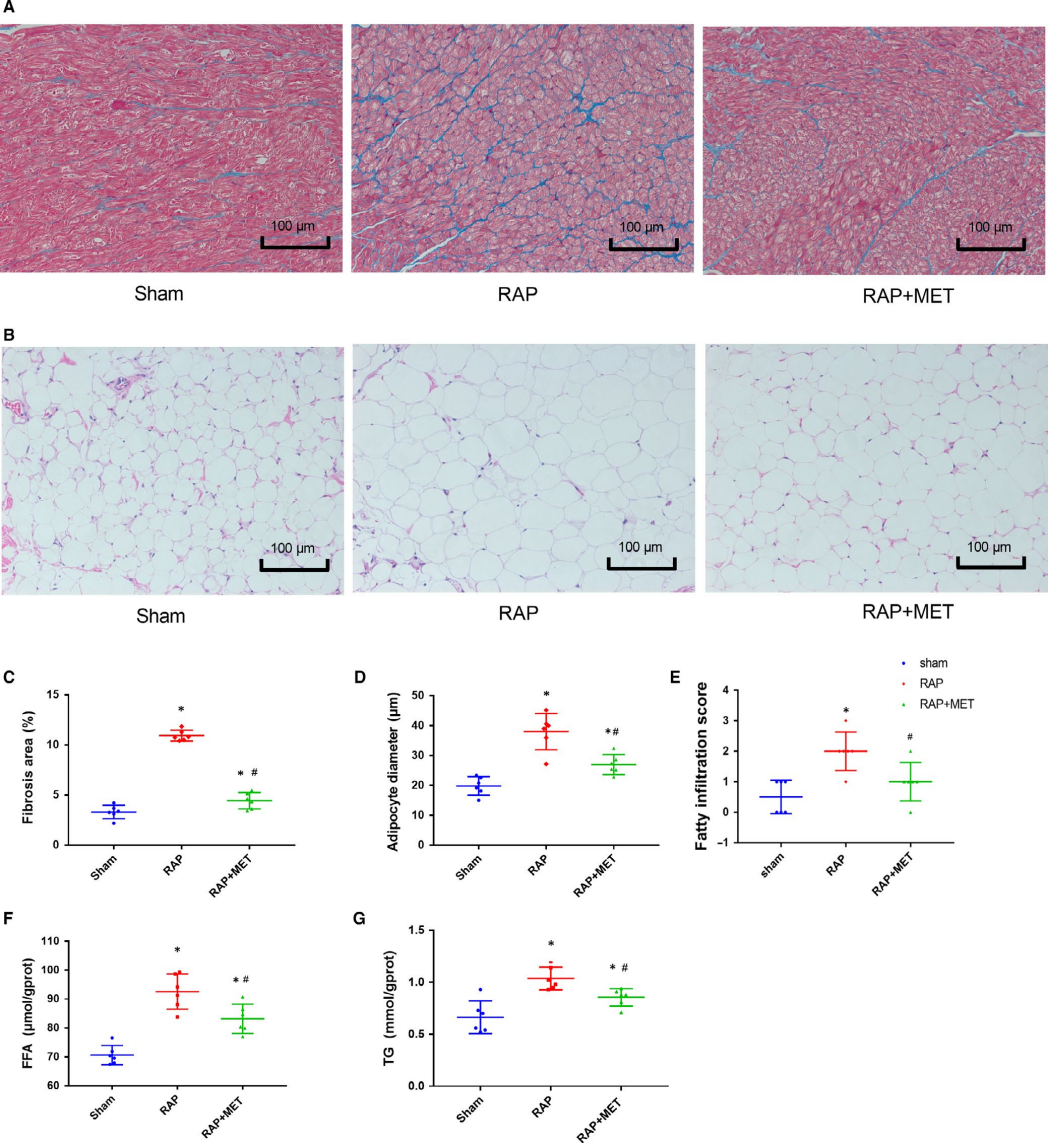
Metformin attenuated RAP‐induced atrial fibrosis and epicardial fat deposition. A, B, Representative Masson staining of the left atrium (LA) and HE staining of the epicardial adipose tissue (EAT) in the sham‐operated group, RAP group and RAP + MET group, 6 weeks after pacemaker implantation (n = 6 animals/group, 200 × magnification, scale bar = 100 μm). C, Percentage area of fibrosis in the LA. D, Mean adipocyte Feret's diameter in the EAT. E, Epicardial adipose tissue infiltration score in the sham‐operated group, RAP group and RAP + MET group. F, G, FFA and TG concentrations in the EAT, 6 weeks after pacemaker implantation (n = 6 for each group). ^*^ *P* < .05 compared with the sham‐operated group; ^#^ *P* < .05 compared with the RAP group. FFA, free fatty acid; TG, triglyceride

Masson staining revealed an increase in interstitial fibrosis in the LA after 6‐week RAP compared to that in the sham‐operated group (10.9% ± 0.5% vs. 3% ± 0.6%, *P* < .01). MET significantly reduced atrial fibrosis (10.9% ± 0.5% to 4.4% ± 0.8%, *P* < .01) (Figure 2A,C).

In the sham‐operated group, no or mild adipose infiltration was observed, whereas in the RAP group, EAT hyperplasia resulted in abundant adipose tissue infiltration accompanied by fibrosis infiltration. MET reversed adipose‐fibrosis infiltration (mean grade: RAP 2 ± 0.67 vs sham 0.5 ± 0.54; RAP 2 ± 0.67 vs RAP + MET 0.83 ± 0.75; both *P* < .05) (Figure 2E). Representative images of EAT from a gross specimen of canine heart and HE‐stained sections demonstrated fatty infiltration at the left posterior atrial wall (Figures S2 and S3).

HE staining showed that adipocytes from the three EAT groups had different cell morphologies (Figure 2B,D). Adipocytes from RAP EAT had a fatter morphology and larger size than the sham‐operated group, whereas MET decreased EAT adipocyte size. The FFA and TG levels in EAT were significantly higher (*P* < .01) in the RAP group than that in the sham‐operated group; and MET effectively decreased the FFA and TG levels in EAT (*P* = .01 and *P* = .04, respectively) (Figure 2F,G).

### 3.3 Effect of metformin on the profiles of cytokines

**Figure 3:**
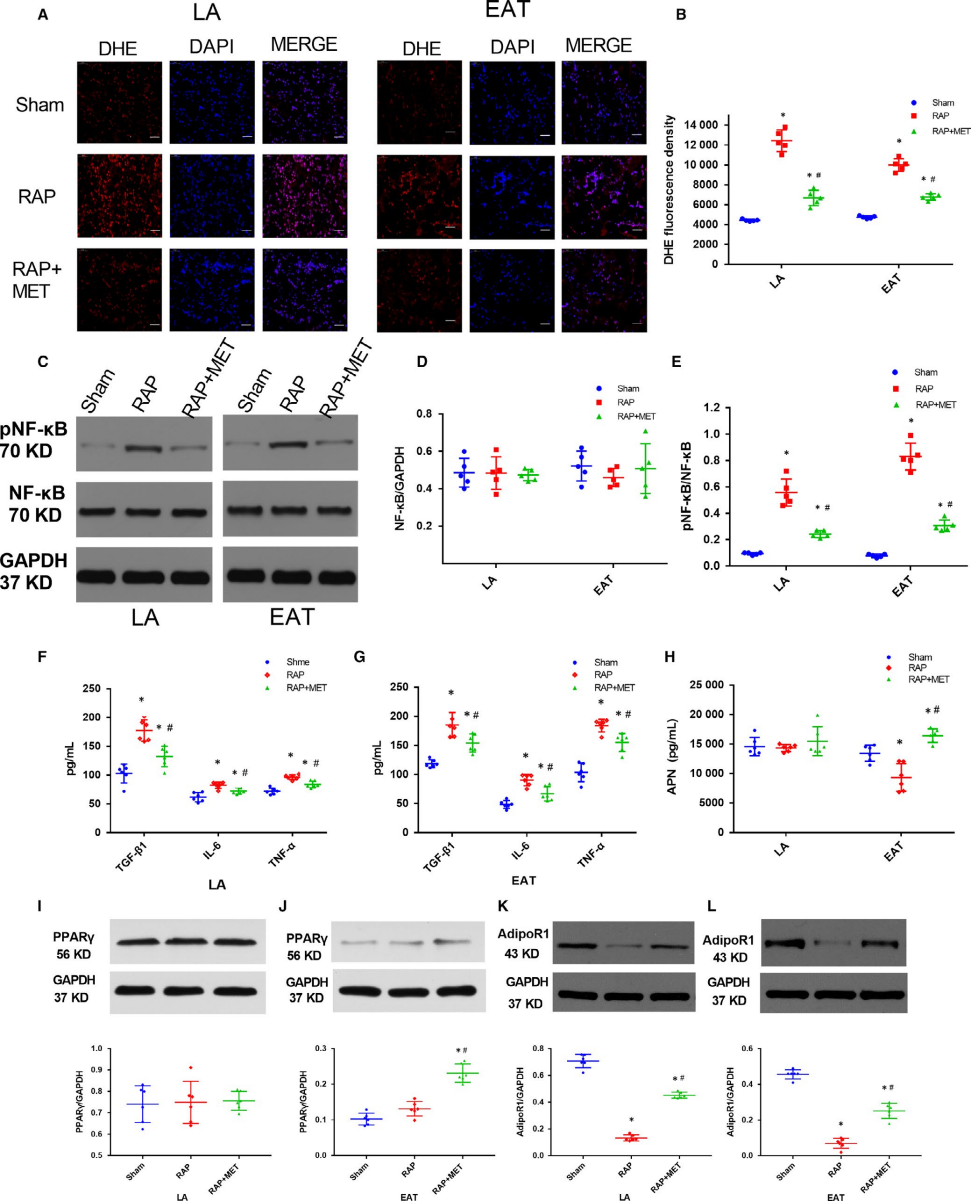
Cytokines and APN system key factors altered in response to RAP and metformin treatment. A, B, Representative DHE staining and quantitative analysis to detect ROS in the LA and in the EAT in the sham‐operated group, RAP group and RAP + MET group, 6 weeks after pacemaker implantation (n = 6 animals/group, 400 × magnification, scale bar = 50 μm). C‐E, Representative immunoblots and quantitative analysis of the relative changes in NF‐κB and pNF‐κB expression in the LA and the EAT. F‐H, TGF‐β1, IL‐6, TNF‐α, and APN concentrations in the LA and EAT in the sham‐operated group, RAP group and RAP + MET group, 6 weeks after surgery (n = 6 animals/group). I‐L, Representative immunoblots and quantitative analysis of the relative changes in PPARγ and AdipoR1 in the LA and EAT. ^*^ *P* < .05 compared with the sham‐operated group; ^#^ *P* < .05 compared with the RAP group. DHE, dihydroethidium; LA, left atrium; EAT, epicardial adipose tissue; TGF‐β1, transforming growth factor‐β1; IL‐6, interleukin‐6; TNF‐α, tumour necrosis factor‐α; APN, adiponectin

AF is closely related to oxidative stress and inflammation, and EAT is an active endocrine organ that produces many cytokines; thus, plays an important role in inflammation of the adjacent myocardium. ROS accumulation and activation of specific redox‐sensitive signalling pathways contribute to AF progression. The ROS content in LA and EAT was significantly higher in the RAP group than that in the sham group, and MET treatment inhibited ROS generation (*P* < .05) (Figure 3A,B). The redox‐sensitive nuclear factor kappa‐B (NF‐κB) signalling pathway was activated after RAP. Although NF‐κB expression was not significantly different among the three groups (*P* > .05) (Figure 3C,E), the phosphorylation level of NF‐κB was significantly higher in the RAP group than in the other two groups both in the LA and EAT (*P* < .05) (Figure 3C,D). Consistent with the alterations in ROS content, MET suppressed NF‐κB signalling activation and decreased the phosphorylation level of NF‐κB in LA and EAT (*P* < .05) (Figure 3C,D).

Next, we detected the concentration of inflammatory adipokines (IL‐6, TNF‐α and TGF‐β1) in the EAT, LA and plasma of each group. RAP significantly increased IL‐6, TNF‐α and TGF‐β1 concentration in EAT and LA; MET attenuated the RAP‐induced expression of IL‐6, TNF‐α and TGF‐β1 (*P* < .05) (Figure 3F,G). In contrast, RAP significantly reduced the anti‐inflammatory adipokine APN level in EAT (13 452.7 ± 1365.3 pg/mL to 9350.3 ± 2356.3 pg/mL, *P* < .01) (Figure 3H), and MET increased APN protein level in EAT compared to that in the RAP group (16 437.8 ± 1156.9 vs 9350.3 ± 2356.3 pg/mL, *P* < .01). No significant difference in APN level in the LA among the three groups was observed, even though MET tended to increase the APN level in the LA (*P* > .05) (Figure 3H). At the baseline, before pacemaker implantation, IL‐6, TNF‐α, TGF‐β1 and APN plasma concentration did not significantly differ among the three groups (*P* > .05). Compared to the plasma concentration in the sham‐operated group, after 6‐week RAP, IL‐6 plasma concentration increased and APN plasma concentration decreased (IL‐6, 43.83 ± 11.41 vs 110.25 ± 24.58 pg/mL, *P* < .01; APN, 532.02 ± 55.39 vs 166.34 ± 51.48 pg/mL, *P* < .01); MET reversed these changes (IL‐6 110.25 ± 24.58 vs. 80.25 ± 19.25 pg/mL, *P* < .01; APN 166.34 ± 51.48 vs. 447.33 ± 53.67 pg/mL, *P* < .01) (Figure S4A,C). TNF‐α and TGF‐β1 plasma concentration showed no significant difference among the three groups after 6 weeks (Figure S4B,D).

### 3.4 Effect of metformin on key factors related to APN production and function

APN expression is highly correlated with the regulation of PPARγ, a key transcriptional factor controlling adipokine genes expression.^25^ Similar to the APN concentration in the LA (Figure 3H), PPARγ protein expression in the LA showed no difference among the three groups (*P* > .05) (Figure 3I).There was no significant difference in PPARγ expression in the EAT between the sham‐operated and RAP groups (*P* > .05), but MET significantly up‐regulated PPARγ protein expression (*P* < .01) (Figure 3J), indicating increased PPARγ/APN production in EAT by MET.

AdipoR1 is one of the three receptors of APN and is the primary APN receptor isoform in skeletal/cardiac muscles. AdipoR1 protein expression was significantly reduced after 6‐week RAP in both LA and EAT (*P* < .01). MET attenuated the RAP‐induced decrease of AdipoR1 expression in LA and EAT (*P* < .01; Figure 3K,L). In addition, mRNA levels of PPARγ and APN in EAT and AdipoR1 in the LA were significantly decreased in the RAP group and these alterations were inhibited by MET (Figure S5A‐C). APN signalling in the LA and EAT was impaired in the RAP group because of the lower AdipoR1 expression. To detect the direct effects of MET on cardiomyocytes, HL‐1 cells were incubated with or without 4 mmol/L MET for 12 hours and exposed to 1 µg/mL LPS for 24 hours. The expression of PPARγ, APN and adipoR1 showed no significant difference among the four groups (Figure S6A,C).

Based on these results, the biological functions of APN, including the anti‐inflammatory and cardioprotective effects, were limited by RAP, while MET enhanced the cardioprotective effects of APN during AF by augmenting APN production in EAT and AdipoR1 expression in EAT and LA.

### 3.5 Metformin suppressed the dysregulation of adipokines secretion in LPS‐ stimulated 3T3‐L1 adipocytes

The effects of MET on APN production, its anti‐inflammation potential and the interaction between atrial muscle and EAT were observed in vivo. We evaluated the protective effect of MET against LPS‐stimulated 3T3‐L1 adipocytes inflammation in vitro. After treating 3T3‐L1 adipocytes with LPS, the APN and TNF‐α concentrations in supernatants were detected by ELISA.

**Figure 4:**
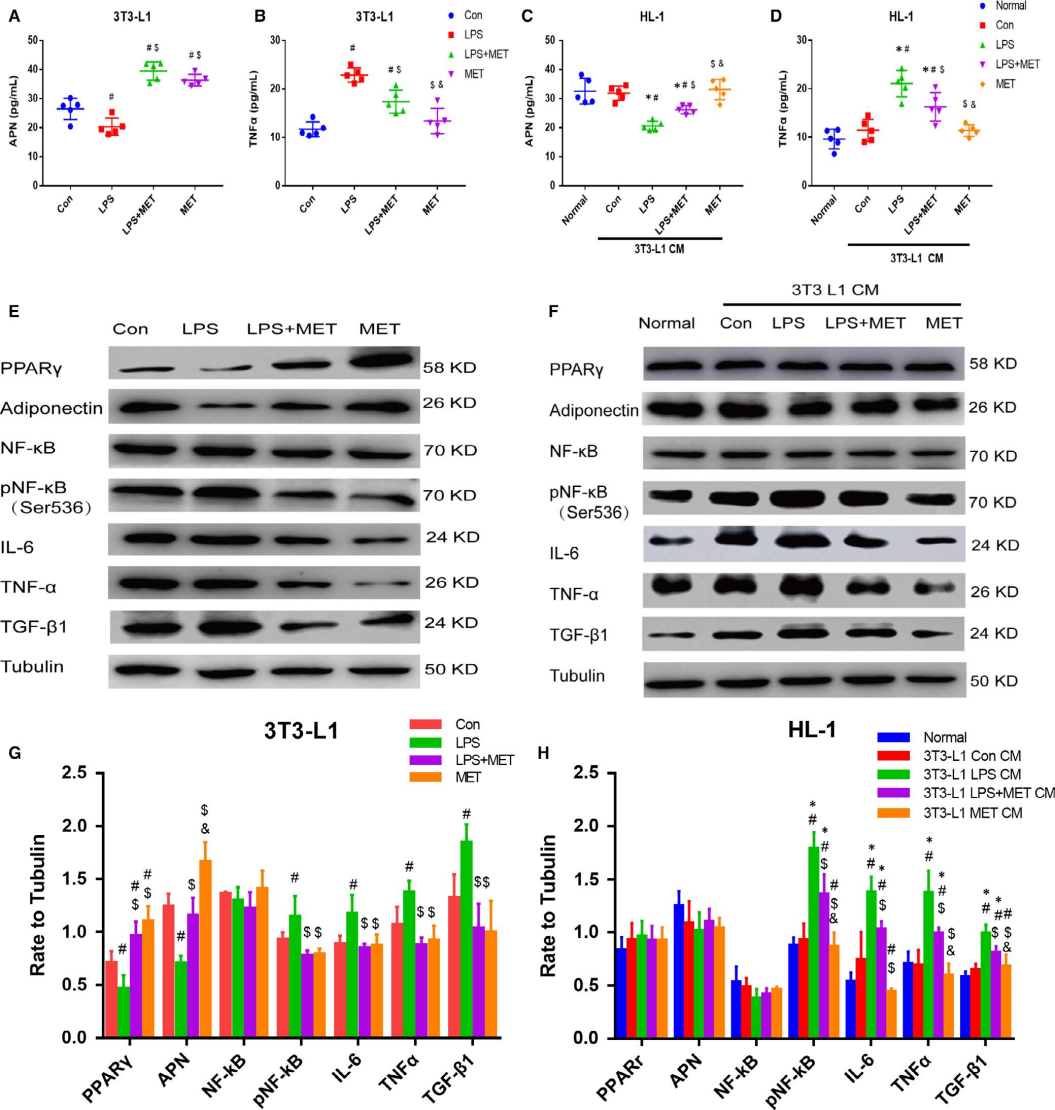
Metformin suppressed LPS‐stimulated inflammation in 3T3‐L1 adipocytes and supernatant‐mediated inflammation in HL‐1 cardiomyocytes. A‐D, APN and TNF‐α concentrations in HL‐1 and 3T3‐L1 supernatants after the indicated experiments. All experiments have been repeated 5 times (n = 5). E‐H, Representative immunoblots and quantitative analysis of the relative changes in intracellular inflammatory factors expression in 3T3‐L1 adipocytes and HL‐1 myocytes. All experiments have been repeated 5 times (n = 5). ^*^ *P* < .05 compared with the normal group; ^#^ *P* < .05 compared with the control group or 3T3‐L1 Con CM group, ^$^ *P* < .05 compared with the LPS group or 3T3‐L1 LPS CM group, ^&^ *P* < .05 compared with the LPS + MET group or 3T3‐L1 LPS + MET CM group

Compared to the concentration in the control group, APN concentration in 3T3‐L1 supernatants decreased and TNF‐α concentration increased in the LPS group (*P* < .05; Figure 4A,B). MET significantly increased APN concentration in the LPS + MET and MET groups compared to that in the control and LPS groups (*P* < .05) (Figure 4A). TNF‐α concentration in the LPS + MET group was lower than that in the LPS group but higher than that in the control group (*P* < .05) (Figure 4B).

Western blotting was used to evaluate intracellular protein expression. As shown in Figure 4E, the level of phospho‐NF‐κB p65 (Ser536) and expression of IL‐6, TNF‐α and TGF‐β1 were significantly increased in the LPS group while PPARγ/APN expression were decreased, compared to those in the control group (*P* < .05) (Figure 4E,G). Pre‐treatment with MET reversed the LPS‐stimulated inflammation response and significantly up‐regulated PPARγ/APN expression (*P* < .05) (Figure 4E,G). As shown in Figure S6B,D, to test the effects of MET treatment initiation after LPS stimulation, 3T3‐L1 cells were exposed to 1 μg/mL LPS for 6 hours prior to MET treatment for 24 hours. MET reversed the LPS‐stimulated up‐regulation of IL‐6, TNF‐α and TGF‐β1 and significantly up‐regulated PPARγ/APN expression. The anti‐inflammatory effect of MET was similar to that of MET treatment prior to LPS exposure. These results indicated that MET is capable of increasing PPARγ/APN expression and suppressing the LPS‐stimulated adipocyte inflammation response, which was consistent with its anti‐inflammatory effect in vivo.

### 3.6 Metformin attenuated the inflammatory interaction between HL‐1 cardiomyocytes and 3T3‐L1 adipocytes

To detect the effect of MET on the inflammatory interaction between adipocytes and cardiomyocytes, HL‐1 was indirectly co‐cultured with 3T3‐L1 as described in method part. The intracellular and supernatant cytokine levels of HL‐1 cells were determined after 48 hours of 3T3 L1 CM treatment. APN concentration in HL‐1 supernatants significantly decreased, and TNF‐α concentration increased under LPS CM compared to the normal and control CM groups (*P* < .05) (Figure 4C,D). When HL‐1 cells were treated with LPS + MET CM and MET CM, the supernatant concentration of APN was higher and TNF‐α concentration was lower than those cells which were exposed to the LPS CM treatment. In addition, APN concentration was even higher, and TNF‐α concentration was even lower in the MET CM group (*P* < .05) (Figure 4C,D).

There was no significant difference in HL‐1 intracellular PPARγ and APN expression among all treatment groups (*P* > .05) (Figure 4F,H). However, intracellular protein levels of phospho‐NF‐κB p65 (Ser536), IL‐6, TNF‐α and TGF‐β1 under LPS CM were significantly higher than those in the other treatment groups (*P* < .05) (Figure 4F,H). Inflammatory cytokine levels under LPS + MET CM were significantly lower than those in the LPS CM group, but were still higher than those in the normal medium and control CM groups (*P* < .05). Phospho‐NF‐κB p65 (Ser536), IL‐6 and TNF‐α levels in the MET CM group were still lower than those in the Control CM group (*P* < .05). These results demonstrated that MET increases adipocyte APN production and secretion and plays a critical role in suppressing the adipocytes and cardiomyocytes inflammatory interaction.

### 3.7 Effect of 3T3 L1 CM on the Ca^2+^ homeostasis in HL‐1 atrial myocytes

**Figure 5:**
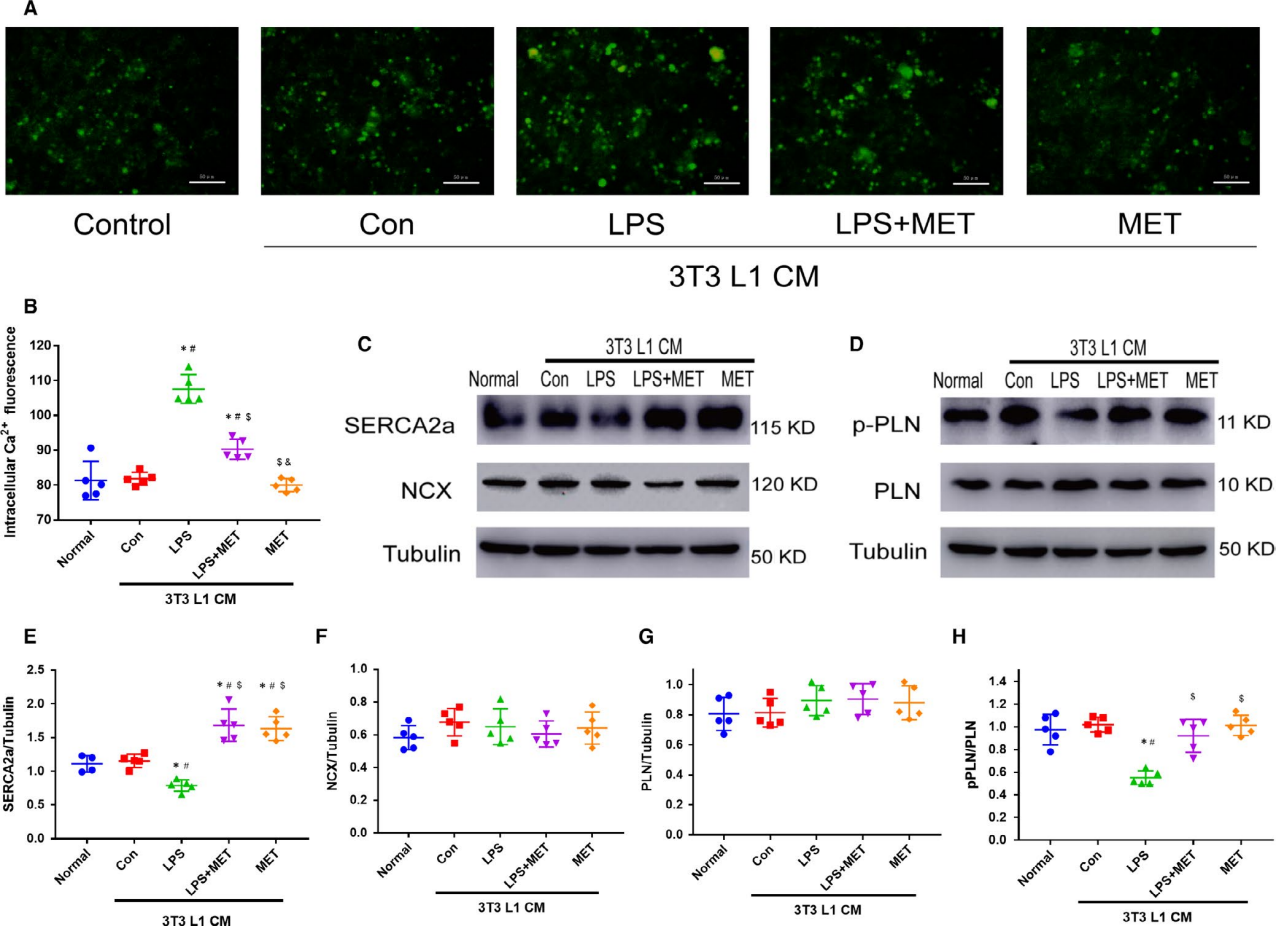
3T3‐L1 CM affected the Ca^2+^ homeostasis in HL‐1 atrial myocytes. A, Representative image of HL‐1 cytosolic Ca^2+^, measured with Fluo‐4 AM following 3T3‐L1 CM treatment. B, Quantitative analysis of intracellular Ca^2+^ fluorescence, using a fluorescent microplate reader. All experiments have been repeated 5 times (n = 5). C‐H, Representative immunoblots and quantitative analysis of the relative changes in HL‐1 Ca‐handling proteins after 3T3‐L1 CM treatment. All experiments have been repeated 5 times (n = 5). ^*^ *P* < .05 compared with the normal group; ^#^ *P* < .05 compared with the control group, ^$^ *P* < .05 compared with the LPS group, ^&^ *P* < .05 compared with the LPS + MET group

Cytosolic Ca^2+^ homeostasis is critical for cardiomyocyte function. To detect the effect of adipokines on Ca^2+^ homeostasis in HL‐1 atrial myocytes, the cytosolic Ca^2+^ of HL‐1 cells were detected with Fluo‐4 AM after 3T3‐L1 CM treatment. Cytosolic Ca^2+^ content was indicated by Fluo‐4 AM fluorescence intensity. HL‐1 cells treated with LPS CM showed cytosolic Ca^2+^ overloading with the highest fluorescence intensity among the four groups (*P* < .05) (Figure 5A,B). Compared to the normal group, there was no significant difference in fluorescence intensity under the other 3T3‐L1 CM (*P* > .05) (Figure 5A,B).

We then detected part of the expression of the Ca^2+^ handlings that were responsible for regulating the Ca^2+^ homeostasis in HL‐1 cells. The sodium‐calcium exchanger (NCX) is mainly responsible for Ca^2+^ extruding from the cell to prevent overloading of intracellular stores. Phospholamban （PLN） is an inhibitory protein that controls the reuptake of Ca^2+^ into the sarcoplasmic reticulum(SR) by the sarcoplasmic reticulum Ca^2+^‐ATPase2a (SERCA2a). Phosphorylation of PLN dissociates the molecule from SERCA and stimulates the reuptake of Ca^2+^ into the SR. As shown in Figure 5C, there was no significant difference in the protein expression of NCX and PLN among the five groups (Figure 5C,E,F) (*P* > .05), while the phosphorylation level of PLN and SERCA2a expression were significantly decreased under LPS CM (*P* < .05, respectively) (Figure 5C,D,G). In addition, SERCA2a expression under LPS + MET CM and MET CM treatments was significantly up‐regulated compared to that in the LPS CM groups (*P* < .05, respectively) (Figure 5C,D). These data indicated that excess cytosolic Ca^2+^ in HL‐1 induced by LPS CM may result from reduced reuptake of cytosolic Ca^2+^ into the SR. The 3T3‐L1 released factors may play an important role in regulating Ca^2+^ handling protein expression and Ca^2+^ homeostasis in HL‐1 atrial myocytes.

### 3.8 Effect of APN on the inflammatory response and the Ca^2+^ homeostasis in HL‐1 atrial myocytes

**Figure 6:**
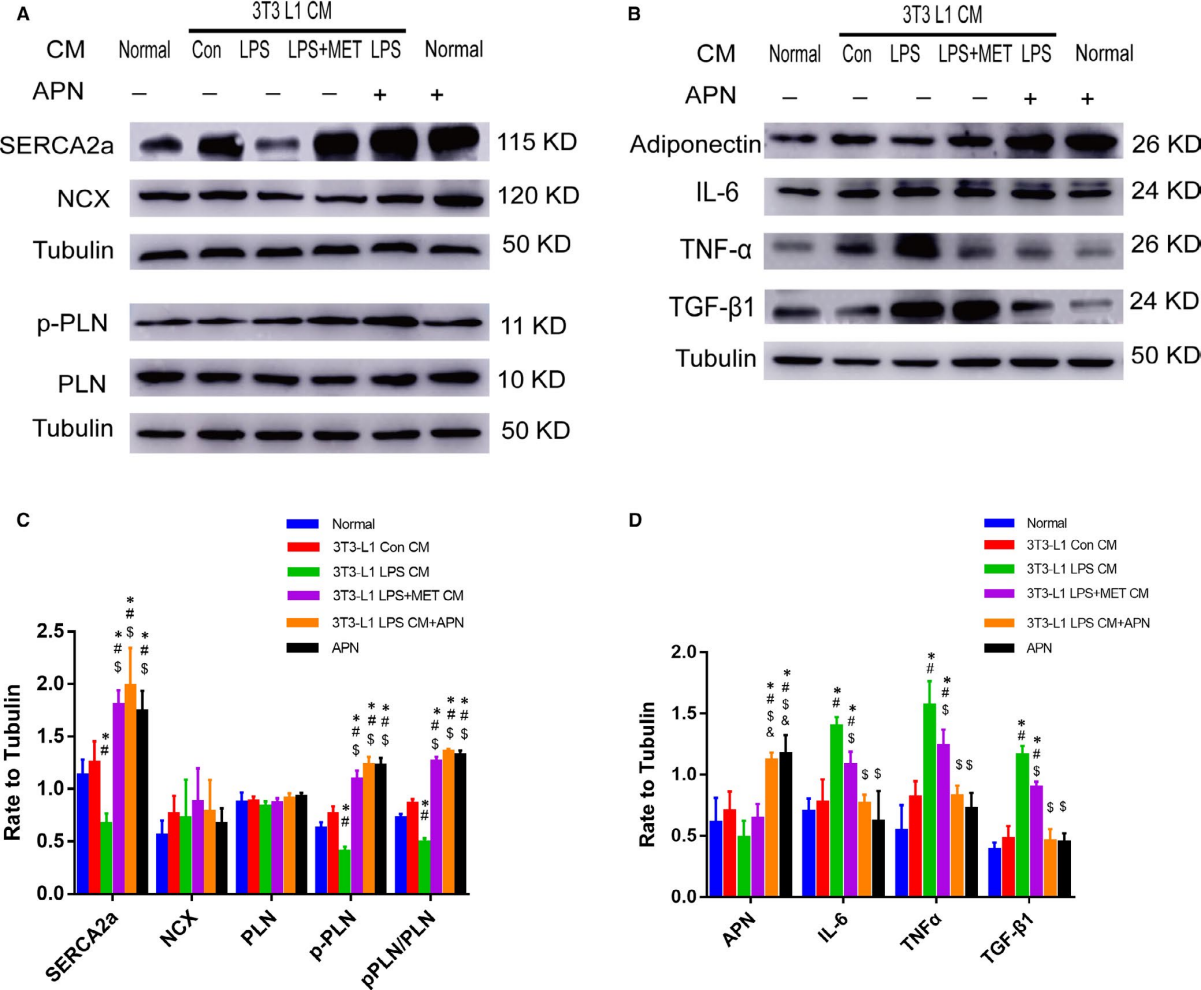
APN treatment reduced the expression of inflammatory factors and improved the function of sarcoplasmic reticulum Ca^2+^ handling. A, C, Representative immunoblots and quantitative analysis of the relative changes in Ca^2+^ handling protein expression in HL‐1 after 3T3‐L1 CM and APN treatment. All experiments have been repeated 5 times (n = 5). B, D, Representative immunoblots and quantitative analysis of inflammatory factors and APN expression in HL‐1 cells after 3T3‐L1 CM and APN treatment. All experiments have been repeated 5 times (n = 5). ^*^ *P* < .05 compared with the normal group; ^#^ *P* < .05 compared with the 3T3‐L1 Con CM group, ^$^ *P* < .05 compared with the 3T3‐L1 LPS CM group

The APN concentration in 3T3‐L1 LPS CM was lower than that in LPS + MET CM and MET CM. LPS CM treatment increased the inflammatory cytokine expression in HL‐1 cells and disturbed Ca^2+^ homeostasis. To determine whether the decrease in APN contributed to atrial myocyte inflammatory response and calcium dyshomeostasis, APN (1 µg/mL) was added when HL‐1 was treated with 3T3‐L1 LPS CM. As shown in Figure 6, the effect of APN treatment was similar to that of LPS + MET CM treatment. In term of Ca^2+^ handling, APN reversed the decrease in PLN phosphorylation and SERCA2a down‐regulation induced by LPS CM (Figure 6A,C) (*P* < .05, respectively). In term of inflammatory cytokine expression, APN attenuated LPS CM treatment induced IL‐6, TNF‐α, and TGF‐β1 up‐regulation (Figure 6B,D) (*P* < .05, respectively).

**Figure 7:**
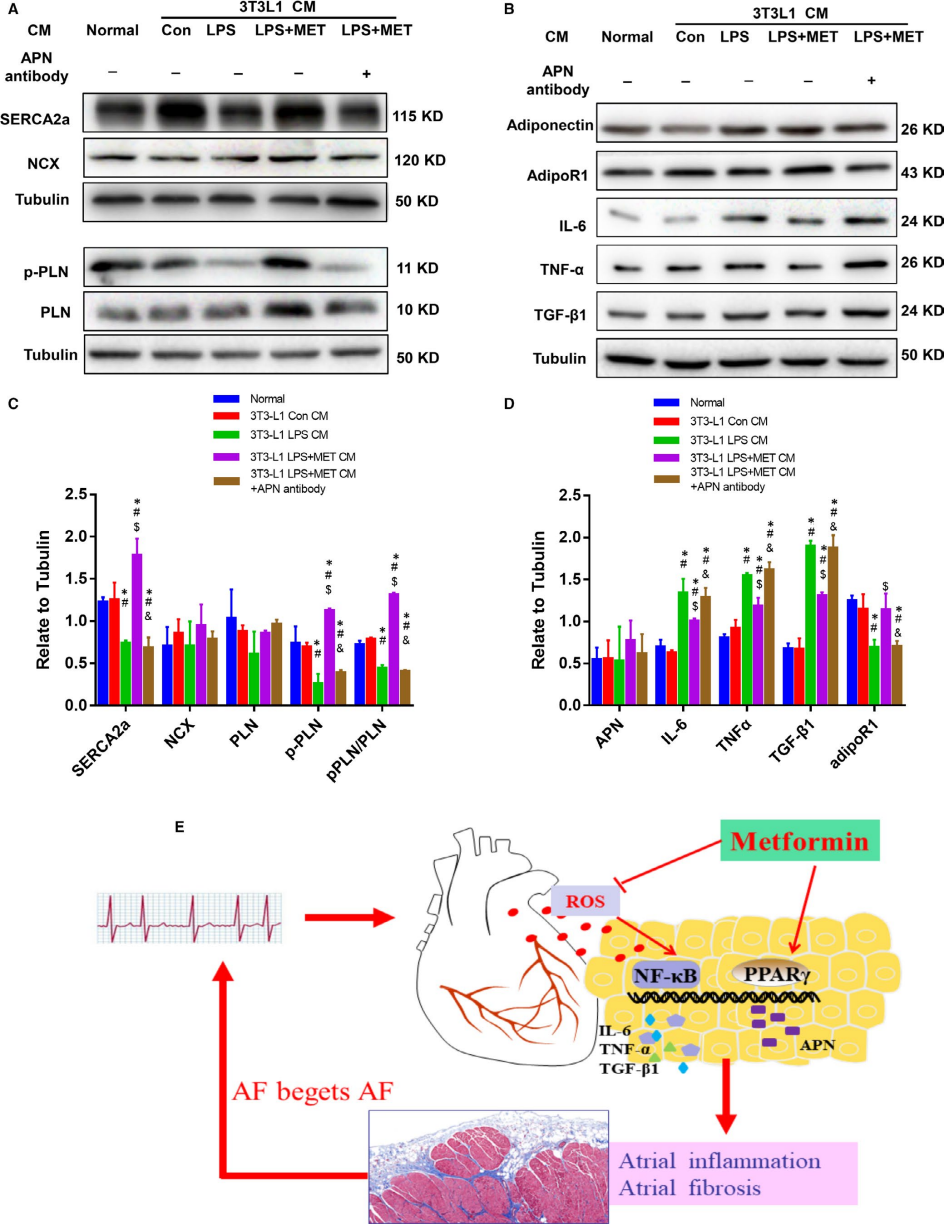
Pre‐neutralizing APN blocked its effect on inflammatory response and Ca^2+^ homeostasis. A, C, Representative immunoblots and quantitative analysis of the relative changes in Ca‐handling protein expression in HL‐1 cells after treatment with 3T3‐L1 CM or 3T3‐L1 LPS + MET CM, which was pre‐neutralized using an adiponectin antibody. All experiments have been repeated 3 times (n = 3). B, D, Representative immunoblots and quantitative analysis of the relative changes in inflammatory factors and APN in HL‐1 cells after treatment with 3T3‐L1 CM or 3T3‐L1 LPS + MET CM, which was pre‐neutralized using an adiponectin antibody. ^*^ *P* < .05 compared with the normal group; ^#^ *P* < .05 compared with the 3T3‐L1 Con CM group, ^$^ *P* < .05 compared with the 3T3‐L1 LPS CM group, ^&^ *P* < .05 compared with the 3T3‐L1 LPS + MET CM group. E, Metformin reversed atrial fibrillation‐induced epicardial adipose tissue (EAT) remodelling, which in turn contributed to AF progression. Atrial fibrillation led to epicardial adipose tissue remodelling and promoted atrial inflammation and fibrosis. Metformin suppressed the ROS/NF‐κB signalling pathway and reduced EAT inflammatory cytokine secretion. In addition, MET activated the PPARγ/APN signalling pathway, further attenuated adjacent myocardial inflammation and fibrosis to interrupt the vicious circle of ‘AF begets AF’

In addition, the effects of APN in 3T3‐L1 CM were demonstrated by neutralizing the condition medium with an anti‐adiponectin antibody. As Figure 7A‐D shows, the effects of LPS + MET CM on Ca^2+^ handling and inflammatory cytokine expression were reversed when the LPS + MET CM was pre‐treated with adiponectin antibody. These results indicated that APN plays a key role in anti‐inflammatory response and the maintenance of Ca^2+^ homeostasis.

## 4 DISCUSSION

### 4.1 Main findings

First, as shown in Figure 7E, we demonstrated the closely related interactions between EAT remodelling and atrial remodelling in response to RAP. Second, we demonstrated that MET reduced AF vulnerability and atrial fibrosis. Third, we demonstrated that MET inhibited ROS/NF‐κB activation, reduced pro‐inflammatory adipokines (IL‐6, TNF‐α and TGF‐β1) expression in the LA and EAT and up‐regulated PPARγ/APN expression in EAT.

### 4.2 Crosstalk between EAT and myocardium during AF

A complex crosstalk exists between EAT and the neighbouring atrial myocardium during AF.^8^, ^26^ Chilukoti et al indicated that AF and rapid pacing induce the expression of several genes that can up‐regulate adipose tissue accumulation.^5^ In an AF sheep model, total atrial and left atrial adipose tissue volume increases significantly after 16‐week AF induction.^27^ These intriguing findings point to the role of AF and rapid pacing in promoting adipocyte differentiation and atrial adipogenesis, and regulating adipose tissue accumulation.^2^, ^5^

Factors derived from EAT play a central role in the development and progression of cardiovascular diseases. Under healthy conditions, APN, omentin‐1 and apelin act on cardiomyocytes in a positive manner, sustaining their regular contractions.^28^ An increased APN level decreases atrial fibrosis and protects against AF development.^29^, ^30^ Pathologically, EAT may mediate deleterious effects on the myocardium.^7^ Adipokines freely diffuse into the adjacent myocardium and may result in fibrotic changes in the atrial myocardium.^7^, ^31^ Pro‐inflammatory cytokines, such as IL‐6 and TNF‐α, are considered key mediators of inflammation‐related atrial fibrillation.^30^, ^32^, ^33^ Fibrotic lesions can also impede electric propagation and slow conduction, increasing AF vulnerability and sustainability.^33^, ^34^ With increasing adiposity, pro‐inflammatory adipokines expression of adipose tissue increase while that of anti‐inflammatory adipokines decrease.^35^

### 4.3 Effect of metformin on adipokines profile and EAT remoulding

RAP induces excess EAT accumulation, which is manifested by a greater EAT adipocyte size and higher FFA and TG concentration in EAT in vivo. Consequently, excess EAT accumulation results in a higher production of inflammatory cytokines and adipokines.^36^, ^37^ Infiltration of macrophages and myofibroblasts in EAT are observed in patients with AF, and pro‐inflammatory and pro‐fibrotic cytokines/chemokines in EAT were positively correlated with LA fibrotic remodelling.^38^

Although MET is widely used as a hypoglycaemic drug, various studies have demonstrated its cardioprotective properties independent of its antihyperglycaemic effect.^39^ Previous studies have shown that MET not only reduces the quantity of epicardial fat,^13^ but also significantly reduces the deleterious adipokines secretion and increases APN secretion.^40^, ^41^ APN, an important adipokine with anti‐inflammation and anti‐oxidation properties,^42^, ^43^ could potentially improve atrial remodelling. Assar et al showed that hypoadiponectinaemia in obese patients leads to a higher incidence of post‐operation AF.^44^ Kourliouros et al found that post‐operation AF is associated with a significant reduction in epicardial APN.^45^ In the present study, MET decreased the concentration of pro‐inflammatory adipokines (IL‐6, TNF‐α and TGF‐β1) and increased anti‐inflammatory adipokine (APN) in EAT.

Many complex molecular pathways may be involved in MET attenuated EAT remodelling. RAP increased ROS accumulation and activated NF‐κB signalling in LA and EPA. ROS accumulated in EAT may be transferred from the adjacent myocardium and excess EAT itself increased ROS production. MET repressed ROS/NF‐κB signalling in LA and EAT and attenuated RAP‐induced up‐regulated expression of IL‐6, TNF‐α and TGF‐β1. The anti‐inflammatory potential of MET may be partly due to its modulation of macrophage differentiation and polarization,^9^ which requires further investigation with our model. Conversely, MET also significantly up‐regulated adipocyte PPARγ/APN protein expression in vitro and in vivo, and increased AdipoR1 expression in EAT and LA. A previous study demonstrated that MET treatment results in the up‐regulation of PPARγ expression by modulating the enzyme 12‐lipoxygenase that synthesizes the PPARγ ligand. MET also activated AMP‐activated protein kinase (AMPK) which subsequently up‐regulates PPARγ.^46^ PPARγ exerts anti‐inflammatory effects by antagonizing NF‐κB activation and reduces ROS production,^47^ which may partly contribute to the cardioprotective effect of MET. Additionally, MET has the potential to enhance APN production in adipose tissue, and its receptor expression in muscle and adipose tissues.^15^, ^48^, ^49^ A previous study reported that APN exhibits a dose dependent induction of adipoR1 in vitro.^50^ Consistent with our results, APN was reported to negatively regulate inflammatory response.^51^, ^52^

APN may have an impact on cytosolic Ca^2+^ homeostasis. 3T3‐L1 LPS CM with a low APN concentration induced SERCA2a and p‐PLN (the key proteins responsible for Ca^2+^ reuptake by SR) down‐regulation and inflammatory cytokine up‐regulation in HL‐1 cells. However, 3T3‐L1 LPS + MET CM with a high APN concentration or APN treatment reversed these alterations. A previous study demonstrated that APN decreases the expression of inflammation response genes and increases the expression of SERCA2a in H9C2 cells.^53^ Komei et al observed that chronic APN overexpression improves SERCA2a dysfunction by promoting phosphorylation of PLN in heart failure.^54^ SERCA2a expression and SR Ca^2+^ reuptake decrease during AF.^55^ APN may be able to reverse this change. The in‐depth understanding of the regulation of cytosolic Ca^2+^ homeostasis by APN may provide valuable information to treat AF.

MET may regulate the interaction between EAT and the atrial myocardium through regulating APN production. Interestingly, MET seems to exert minute effects on PPARγ/APN expression in cardiomyocytes. PPARγ in the adipose tissue may, therefore, respond better to MET in the myocardium because of tissue specificity. Therefore, we inferred that the cardioprotective effects of APN mainly arise from the adipose tissue and not from the myocardium. MET not only exhibits an anti‐inflammatory effect by inhibiting ROS/NF‐κB signalling during AF, but also via the APN system.

### 4.4 Metformin reduces atrial electric remodelling and AF inducibility

Atrial remodelling is critical for the pathogenesis of atrial fibrillation.^56^ MET not only reversed RAP‐induced EAT and atrial pro‐inflammatory remodelling, but reversed ERP decrease and AF inducibility. Interstitial fibrosis and adipose infiltration can reduce electrical coupling between cardiomyocytes and impede the electrical conduction of cardiomyocytes. Besides fibrosis, the anti‐arrhythmic effect of MET may alter ionic channels and pump properties. MET is associated with a decrease in corrected QT interval.^57^ It restores the electrophysiology of small conductance calcium‐activated potassium channels in the atrium of Goto Kakizaki diabetic rats, supporting a potential beneficial effect on atrial electrophysiology.^58^ The effect of MET on ion channels of myocardial cells and arrhythmia lack large‐scale clinical studies. The effects of MET on arrhythmia also require further observation.

In addition, MET can modulate several signalling pathways including AMP‐activated protein kinase signalling (AMPK), which has a potential role in cardiac electrophysiology and arrhythmia.^59^ As AF pathology partly contributes to the metabolic change, AMPK activation appears to mediate the metabolic change in irregularly paced myocytes,^60^ but whether MET reduces AF inducibility via AMPK activation requires further investigation.

### 4.5 Study limitations

This study had several limitations. Firstly, although HL‐1 cells are the most commonly used atrial cardiomyocyte cell line, HL‐1 cells represent artificial cardiomyocytes. Secondly, the effect of MET on the cellular and molecular electrophysiology of cardiomyocytes was of interest but beyond the scope of the study; thirdly, there was a lack of structural and hemodynamic data from cardiac MRI scans and the EAT volume was not measured; finally, the operation procedures may have impacted the concentration of inflammatory mediators and AF inducibility. However, as the operation procedure and operation time were similar among the three groups, our data were comparable.

## 5 CONCLUSION

RAP resulted in increased AF vulnerability and atrial remodelling, accompanied by remodelling of EAT adipokines production profiles. AF‐dependent EAT remodelling may occur, at least partially contributing to AF susceptibility. MET reduced AF vulnerability and atrial fibrosis, up‐regulated PPARγ/APN expression in EAT and down‐regulated pro‐inflammatory adipokines expression in LA and EAT. MET may be a potential therapeutic option to prevent and treat AF.

## CONFLICT OF INTEREST

The authors report no relationship that could be construed as a conflict of interest.

## AUTHOR CONTRIBUTIONS

Biao Li and Qiming Liu planned and designed the experiments. Biao Li, Baojian Zhang, Fan Bai and Jiayi Li, and Fen Qin conducted the experiments. Biao Li, Na Liu, Chao Sun, Yichao Xiao and Tao Tu collected and interpreted the data. Biao Li drafted the article, Sunny S. Po, Qiming Liu and Shenghua Zhou revised the manuscript.

## DATA AVAILABILITY STATEMENT

The data that support the findings of this study are available from the corresponding author upon reasonable request.

## Supporting information

Supplementary Material

## References

[1] Benjamin EJ , Wolf PA , D'Agostino RB , Silbershatz H , Kannel WB , Levy D . Impact of atrial fibrillation on the risk of death: the Framingham Heart Study. Circulation. 1998;98:946‐952. 9737513 10.1161/01.cir.98.10.946

[2] Al Chekakie MO , Welles CC , Metoyer R , et al. Pericardial fat is independently associated with human atrial fibrillation. J Am Coll Cardiol. 2010;56:784‐788. 20797492 10.1016/j.jacc.2010.03.071

[3] Wong CX , Abed HS , Molaee P , et al. Pericardial fat is associated with atrial fibrillation severity and ablation outcome. J Am Coll Cardiol. 2011;57:1745‐1751. 21511110 10.1016/j.jacc.2010.11.045

[4] Wong CX , Ganesan AN , Selvanayagam JB . Epicardial fat and atrial fibrillation: current evidence, potential mechanisms, clinical implications, and future directions. Eur Heart J. 2017;38:1294‐1302. 26935271 10.1093/eurheartj/ehw045

[5] Chilukoti RK , Giese A , Malenke W , et al. Atrial fibrillation and rapid acute pacing regulate adipocyte/adipositas‐related gene expression in the atria. Int J Cardiol. 2015;187:604‐613. 25863735 10.1016/j.ijcard.2015.03.072

[6] Abed HS , Samuel CS , Lau DH , et al. Obesity results in progressive atrial structural and electrical remodeling: implications for atrial fibrillation. Heart Rhythm. 2013;10:90‐100. 23063864 10.1016/j.hrthm.2012.08.043

[7] Venteclef N , Guglielmi V , Balse E , et al. Human epicardial adipose tissue induces fibrosis of the atrial myocardium through the secretion of adipo‐fibrokines. Eur Heart J. 2015;36:795‐805a. 23525094 10.1093/eurheartj/eht099

[8] Hatem SN , Redheuil A , Gandjbakhch E . Cardiac adipose tissue and atrial fibrillation: the perils of adiposity. Cardiovasc Res. 2016;109:502‐509. 26790475 10.1093/cvr/cvw001

[9] Vasamsetti SB , Karnewar S , Kanugula AK , Thatipalli AR , Kumar JM , Kotamraju S . Metformin inhibits monocyte‐to‐macrophage differentiation via AMPK‐mediated inhibition of STAT3 activation: potential role in atherosclerosis. Diabetes. 2015;64:2028‐2041. 25552600 10.2337/db14-1225

[10] Qi T , Chen Y , Li H , et al. A role for PFKFB3/iPFK2 in metformin suppression of adipocyte inflammatory responses. J Mol Endocrinol. 2017;59:49‐59. 28559290 10.1530/JME-17-0066 PMC5512603

[11] Cameron AR , Morrison VL , Levin D , et al. Anti‐inflammatory effects of metformin irrespective of diabetes status. Circ Res. 2016;119:652‐665. 27418629 10.1161/CIRCRESAHA.116.308445 PMC4990459

[12] Chang SH , Wu LS , Chiou MJ , et al. Association of metformin with lower atrial fibrillation risk among patients with type 2 diabetes mellitus: a population‐based dynamic cohort and in vitro studies. Cardiovasc Diabetol. 2014;13:123. 25106079 10.1186/s12933-014-0123-x PMC4149273

[13] Jonker JT , Lamb HJ , van der Meer RW , et al. Pioglitazone compared with metformin increases pericardial fat volume in patients with type 2 diabetes mellitus. J Clin Endocrinol Metab. 2010;95:456‐460. 19915017 10.1210/jc.2009-1441

[14] Huypens P , Quartier E , Pipeleers D , Van de Casteele M . Metformin reduces adiponectin protein expression and release in 3T3‐L1 adipocytes involving activation of AMP activated protein kinase. Eur J Pharmacol. 2005;518:90‐95. 16039647 10.1016/j.ejphar.2005.06.016

[15] Zulian A , Cancello R , Girola A , et al. In vitro and in vivo effects of metformin on human adipose tissue adiponectin. Obes Facts. 2011;4:27‐33. 21372608 10.1159/000324582 PMC6444565

[16] Sasaki H , Asanuma H , Fujita M , et al. Metformin prevents progression of heart failure in dogs: role of AMP‐activated protein kinase. Circulation. 2009;119:2568‐2577. 19414638 10.1161/CIRCULATIONAHA.108.798561

[17] Morillo CA , Klein GJ , Jones DL , Guiraudon CM . Chronic rapid atrial pacing. Structural, functional, and electrophysiological characteristics of a new model of sustained atrial fibrillation. Circulation. 1995;91:1588‐1595. 7867201 10.1161/01.cir.91.5.1588

[18] Chen M , Zhou X , Liu Q , et al. Left‐sided noninvasive vagus nerve stimulation suppresses atrial fibrillation by upregulating atrial gap junctions in canines. J Cardiovasc Pharmacol. 2015;66:593‐599. 26317165 10.1097/FJC.0000000000000309

[19] Lu Z , Scherlag BJ , Lin J , et al. Atrial fibrillation begets atrial fibrillation: autonomic mechanism for atrial electrical remodeling induced by short‐term rapid atrial pacing. Circ Arrhythm Electrophysiol. 2008;1:184‐192. 19808412 10.1161/CIRCEP.108.784272 PMC2766842

[20] Yu L , Scherlag BJ , Sha Y , et al. Interactions between atrial electrical remodeling and autonomic remodeling: how to break the vicious cycle. Heart Rhythm. 2012;9:804‐809. 22214613 10.1016/j.hrthm.2011.12.023

[21] Lopez‐Pascual A , Lorente‐Cebrian S , Moreno‐Aliaga MJ , Martinez JA , Gonzalez‐Muniesa P . Inflammation stimulates hypoxia‐inducible factor‐1alpha regulatory activity in 3T3‐L1 adipocytes with conditioned medium from lipopolysaccharide‐activated RAW 264.7 macrophages. J Cell Physiol. 2018;234:550‐560. 30071127 10.1002/jcp.26763

[22] Owen MR , Doran E , Halestrap AP . Evidence that metformin exerts its anti‐diabetic effects through inhibition of complex 1 of the mitochondrial respiratory chain. Biochem J. 2000;348(Pt 3):607‐614. 10839993 PMC1221104

[23] Lin YK , Chen YC , Chang SL , et al. Heart failure epicardial fat increases atrial arrhythmogenesis. Int J Cardiol. 2013;167:1979‐1983. 22633668 10.1016/j.ijcard.2012.05.009

[24] Mahajan R , Lau DH , Brooks AG , et al. Electrophysiological, electroanatomical, and structural remodeling of the atria as consequences of sustained obesity. J Am Coll Cardiol. 2015;66:1‐11. 26139051 10.1016/j.jacc.2015.04.058

[25] Antonopoulos AS , Margaritis M , Verheule S , et al. Mutual regulation of epicardial adipose tissue and myocardial redox state by PPAR‐gamma/adiponectin signalling. Circ Res. 2016;118:842‐855. 26838789 10.1161/CIRCRESAHA.115.307856 PMC4772814

[26] Antonopoulos AS , Antoniades C . The role of epicardial adipose tissue in cardiac biology: classic concepts and emerging roles. J Physiol. 2017;595:3907‐3917. 28191635 10.1113/JP273049 PMC5471417

[27] Haemers P , Hamdi H , Guedj K , et al. Atrial fibrillation is associated with the fibrotic remodelling of adipose tissue in the subepicardium of human and sheep atria. Eur Heart J. 2017;38:53‐61. 26612579 10.1093/eurheartj/ehv625

[28] Rietdorf K , MacQueen H . Investigating interactions between epicardial adipose tissue and cardiac myocytes: what can we learn from different approaches? Br J Pharmacol. 2017;174:3542‐3560. 27882550 10.1111/bph.13678 PMC5610165

[29] Burstein B , Nattel S . Atrial fibrosis: mechanisms and clinical relevance in atrial fibrillation. J Am Coll Cardiol. 2008;51:802‐809. 18294563 10.1016/j.jacc.2007.09.064

[30] Conway DS , Buggins P , Hughes E , Lip GY . Prognostic significance of raised plasma levels of interleukin‐6 and C‐reactive protein in atrial fibrillation. Am Heart J. 2004;148:462‐466. 15389233 10.1016/j.ahj.2004.01.026

[31] Mazurek T , Zhang L , Zalewski A , et al. Human epicardial adipose tissue is a source of inflammatory mediators. Circulation. 2003;108:2460‐2466. 14581396 10.1161/01.CIR.0000099542.57313.C5

[32] Smit MD , Maass AH , De Jong AM , Muller Kobold AC , Van Veldhuisen DJ , Van Gelder IC . Role of inflammation in early atrial fibrillation recurrence. Europace. 2012;14:810‐817. 22237586 10.1093/europace/eur402

[33] Thanigaimani S , McLennan E , Linz D , et al. Progression and reversibility of stretch induced atrial remodeling: Characterization and clinical implications. Prog Biophys Mol Biol. 2017;130:376‐386. 28734850 10.1016/j.pbiomolbio.2017.07.010

[34] Verheule S , Sato T , Tt E , et al. Increased vulnerability to atrial fibrillation in transgenic mice with selective atrial fibrosis caused by overexpression of TGF‐beta1. Circ Res. 2004;94:1458‐1465. 15117823 10.1161/01.RES.0000129579.59664.9d PMC2129102

[35] Parker‐Duffen JL , Walsh K . Cardiometabolic effects of adiponectin. Best Pract Res Clin Endocrinol Metab. 2014;28:81‐91. 24417948 10.1016/j.beem.2013.09.001 PMC3905311

[36] Vazquez‐Vela ME , Torres N , Tovar AR . White adipose tissue as endocrine organ and its role in obesity. Arch Med Res. 2008;39:715‐728. 18996284 10.1016/j.arcmed.2008.09.005

[37] Fei J , Cook C , Blough E , Santanam N . Age and sex mediated changes in epicardial fat adipokines. Atherosclerosis. 2010;212:488‐494. 20667540 10.1016/j.atherosclerosis.2010.06.044 PMC2952711

[38] Abe I , Teshima Y , Kondo H , et al. Association of fibrotic remodeling and cytokines/chemokines content in epicardial adipose tissue with atrial myocardial fibrosis in patients with atrial fibrillation. Heart Rhythm. 2018;15:1717‐1727. 29908372 10.1016/j.hrthm.2018.06.025

[39] Varjabedian L , Bourji M , Pourafkari L , Nader ND . Cardioprotection by metformin: beneficial effects beyond glucose reduction. Am J Cardiovasc Drugs. 2018;18:181‐193. 29478240 10.1007/s40256-018-0266-3

[40] Evia‐Viscarra ML , Rodea‐Montero ER , Apolinar‐Jimenez E , et al. The effects of metformin on inflammatory mediators in obese adolescents with insulin resistance: controlled randomized clinical trial. J Pediatr Endocrinol Metab. 2012;25:41‐49. 22570949 10.1515/jpem-2011-0469

[41] Su JR , Lu ZH , Su Y , et al. Relationship of serum adiponectin levels and metformin therapy in patients with type 2 diabetes. Horm Metab Res. 2016;48:92‐98. 26808583 10.1055/s-0035-1569287

[42] Wang ZV , Scherer PE . Adiponectin, cardiovascular function, and hypertension. Hypertension. 2008;51:8‐14. 17998473 10.1161/HYPERTENSIONAHA.107.099424

[43] Essick EE , Ouchi N , Wilson RM , et al. Adiponectin mediates cardioprotection in oxidative stress‐induced cardiac myocyte remodeling. Am J Physiol Heart Circ Physiol. 2011;301:H984‐H993. 21666115 10.1152/ajpheart.00428.2011 PMC3191107

[44] Low AO . adiponectin level may contribute to higher incidence of postcardiac surgery atrial fibrillation in obese patients. Ann Thorac Surg. 2012;93:1762‐1763; author reply 3. 10.1016/j.athoracsur.2011.09.025 22541225

[45] Kourliouros A , Karastergiou K , Nowell J , et al. Protective effect of epicardial adiponectin on atrial fibrillation following cardiac surgery. Eur J Cardiothorac Surg. 2011;39:228‐232. 20609593 10.1016/j.ejcts.2010.05.006

[46] Elia EM , Pustovrh C , Amalfi S , Devoto L , Motta AB . Link between metformin and the peroxisome proliferator‐activated receptor gamma pathway in the uterine tissue of hyperandrogenized prepubertal mice. Fertil Steril. 2011;95: 2534‐2537 e1. 21382619 10.1016/j.fertnstert.2011.02.004

[47] Mansour HH , El Kiki SM , Galal SM . Metformin and low dose radiation modulates cisplatin‐induced oxidative injury in rat via PPAR‐gamma and MAPK pathways. Arch Biochem Biophys. 2017;616:13‐19. 28104448 10.1016/j.abb.2017.01.005

[48] Metais C , Forcheron F , Abdallah P , et al. Adiponectin receptors: expression in Zucker diabetic rats and effects of fenofibrate and metformin. Metabolism. 2008;57:946‐953. 18555836 10.1016/j.metabol.2008.02.010

[49] Asensio‐Lopez MC , Lax A , Pascual‐Figal DA , Valdes M , Sanchez‐Mas J . Metformin protects against doxorubicin‐induced cardiotoxicity: involvement of the adiponectin cardiac system. Free Radic Biol Med. 2011;51:1861‐1871. 21907790 10.1016/j.freeradbiomed.2011.08.015

[50] Dai Y , Pang J , Gong H , Fan W , Zhang TM . Roles and tissue source of adiponectin involved in lifestyle modifications. J Gerontol A Biol Sci Med Sci. 2013;68:117‐128. 22562959 10.1093/gerona/gls131

[51] Ouchi N , Walsh K . Adiponectin as an anti‐inflammatory factor. Clin Chim Acta. 2007;380:24‐30. 17343838 10.1016/j.cca.2007.01.026 PMC2755046

[52] Shibata R , Sato K , Pimentel DR , et al. Adiponectin protects against myocardial ischemia‐reperfusion injury through AMPK‐ and COX‐2‐dependent mechanisms. Nat Med. 2005;11:1096‐1103. 16155579 10.1038/nm1295 PMC2828682

[53] Boddu NJ , Theus S , Luo S , Wei JY , Ranganathan G . Is the lack of adiponectin associated with increased ER/SR stress and inflammation in the heart? Adipocyte. 2014;3:10‐18. 24575364 10.4161/adip.26684 PMC3917927

[54] Tanaka K , Wilson RM , Essick EE , et al. Effects of adiponectin on calcium‐handling proteins in heart failure with preserved ejection fraction. Circ Heart Fail. 2014;7:976‐985. 25149095 10.1161/CIRCHEARTFAILURE.114.001279 PMC4241144

[55] El‐Armouche A , Boknik P , Eschenhagen T , et al. Molecular determinants of altered Ca2+ handling in human chronic atrial fibrillation. Circulation. 2006;114:670‐680. 16894034 10.1161/CIRCULATIONAHA.106.636845

[56] Schotten U , Verheule S , Kirchhof P , Goette A . Pathophysiological mechanisms of atrial fibrillation: a translational appraisal. Physiol Rev. 2011;91:265‐325. 21248168 10.1152/physrev.00031.2009

[57] Najeed SA , Khan IA , Molnar J , Somberg JC . Differential effect of glyburide (glibenclamide) and metformin on QT dispersion: a potential adenosine triphosphate sensitive K+ channel effect. Am J Cardiol. 2002;90:1103‐1106. 12423711 10.1016/s0002-9149(02)02776-5

[58] Fu X , Pan Y , Cao Q , et al. Metformin restores electrophysiology of small conductance calcium‐activated potassium channels in the atrium of GK diabetic rats. BMC Cardiovasc Disord. 2018;18:63. 29636010 10.1186/s12872-018-0805-5 PMC5894224

[59] Harada M , Nattel SN , Nattel S . AMP‐activated protein kinase: potential role in cardiac electrophysiology and arrhythmias. Circ Arrhythm Electrophysiol. 2012;5:860‐867. 22895602 10.1161/CIRCEP.112.972265

[60] Lenski M , Schleider G , Kohlhaas M , et al. Arrhythmia causes lipid accumulation and reduced glucose uptake. Basic Res Cardiol. 2015;110:40. 26018791 10.1007/s00395-015-0497-2

