# Supplementary material for: Metformin regulates adiponectin signalling in epicardial adipose tissue and reduces atrial fibrillation vulnerability
Source: J Cell Mol Med. 2020 May 22;24(14):7751–66. doi: 10.1111/jcmm.15407 (PMC7348162; doi:10.1111/jcmm.15407)
Supplement: Supplementary file 1 — Supplementary Material [file JCMM-24-7751-s001.docx]

-----Supplemental files are intended for publication as a data supplement.

**Metformin regulates adiponectin signaling in epicardial adipose tissue and reduces atrial fibrillation vulnerability**

Biao Li^1^, Sunny S. Po^2^, Baojian Zhang ^1,3^, Fan Bai^1^, Jiayi Li^1^, Fen Qin^1^, Na Liu^1^, Chao Sun^1^, Yichao Xiao^1^, Tao Tu^1^, Shenghua Zhou^1^, Qiming Liu*^1^

^1^Department of Cardiology/Cardiac Catheterization Lab, Second Xiangya Hospital, Central South University, Changsha City, Hunan Province, China

^2^Heart Rhythm Institute and Department of Medicine, University of Oklahoma Health Sciences Center, Oklahoma City, Oklahoma, United States of America

^3^ Department of Cardiology, the Affiliated Chinese Medicine Hospital of Xinjiang Medical University, Urumqi City, Xinjiang Province, China

*Corresponding to:

Q. Liu,

Department of Cardiology

The Second Xiangya Hospital of Central South University

No. 139 Middle Renmin Road, Furong District, Changsha City, Hunan Province 410011, China

E-mail address: qimingliu@csu.edu.cn (Q. Liu)

### Detailed Methods

### Western blot analyses

Protein-extracts of snap-frozen left atrial tissue, EAT, and whole-cell lysates of HL-1 and 3T3-L1 were prepared using standard procedures. Protein concentrations in the supernatants were measured using Bicinchoninic acid (BCA) assay (ASPEN, USA). Proteins were separated on SDS-polyacrylamide gels and transferred to PVDF membranes. The primary antibodies, rabbit monoclonal anti-GAPDH antibody (diluted 1:10000), rabbit polyclonal anti-PPARγ antibody (diluted 1:1000), rabbit polyclonal anti-AdipoR1 antibody (diluted 1:1000; LS Bio), rabbit monoclonal anti-Adiponectin antibody (diluted 1:1000;Abcam), rabbit polyclonal anti-IL-6 antibody (diluted 1:1000; Proteintech), rabbit polyclonal anti-TNF-α antibody (diluted 1:1000; Abclonal), rabbit polyclonal anti-TGF-β1 antibody (diluted 1:1000; Abcam), rabbit monoclonal anti-NF-κB p65 antibody (diluted 1:1000;Abcam), rabbit monoclonal anti-Phospho-NF-κB p65 (Ser536) antibody (diluted 1:1000; CST), mouse monoclonal anti-Tubulin antibody (diluted 1:5000; Proteintech), rabbit monoclonal anti-SERCA2 ATPase antibody (diluted 1:5000; Abcam), rabbit monoclonal anti-Phospholamban antibody (diluted 1:1000; Abcam), rabbit monoclonal anti-Phospholamban (phospho S16) antibody (diluted 1:50000; Abcam), rabbit polyclonal anti-NCX1 antibody (diluted 1:1000; Abcam),followed by secondary goat anti-rabbit antibody or goat anti-mouse antibody (diluted 1:10000, Proteintech) were used. For loading controls, membranes were stripped with stripping buffer (ASPEN, USA) for 10 min at room temperature. Antibody binding was detected with the ECL detection reagent (ASPEN, USA). Bands were quantified with ImageJ software.

### Real-time quantitative PCR

30–50 mg tissues from LA or EAT were homogenized in TRIZOL(Invitrogen, Carlsbad, CA) for extraction of RNA according to the manufacturer’s protocol. Both reverse transcription and quantitative PCR were carried out with Promega kits (Promega, Madison, WI).The StepOne Real-Time PCR (Life technologies, Alameda, CA) was used for real-time qPCR analysis. The primer of gene PPARγ, Adiponectin, GAPDH was synthesized from GeneCreate (GeneCreate, Wuhan, China). The primer sequences used are listed in supplementary Table 2. The amount of each gene was determined and normalized to the amount of GAPDH. The relative expression quantity 2-ΔΔCt value was calculated to compare the differences among groups.

### Supplementary Tables

Supplementary Table 1. Animals' age and body weight before after interventions. There was no statistic difference among three groups.

|  | Sham | RAP | RAP+MET |
| --- | --- | --- | --- |
| Age (month) | 12.83±0.75 | 12.67±0.81 | 13±1.09 |
| Body weight before intervention（Kg） | 8.82±0.60 | 8.72±0.33 | 8.78±0.33 |
| Body weight after  intervention（Kg） | 8.97±0.54 | 8.95±0.27 | 8.83±0.36 |

Supplementary **Table 2. Sequences of primers used for real-time PCR**

| Primer | Forward (5’ →3’) | Reverse (5’ →3’) |
| --- | --- | --- |
| PPARγ | GTGGATTTCTCCAGCATTTCC | GGCTCTTCGTGAGGCTTATTG |
| Adiponectin | ATGTTCCCATTCGCTTTACCA | CATAGGTGAAGAGCATAGCCTTG |
| AdipoR1 | AAACTGGATTATTCAGGGATTGC | CTTAGGAGTGGCAAACCGGT |
| GAPDH | GAAGGTCGGAGTGAACGGATT | CATTTGATGTTGGCGGGATC |

### Supplementary Figures and Figure Legends

**Supplementary** **Figure 1.**


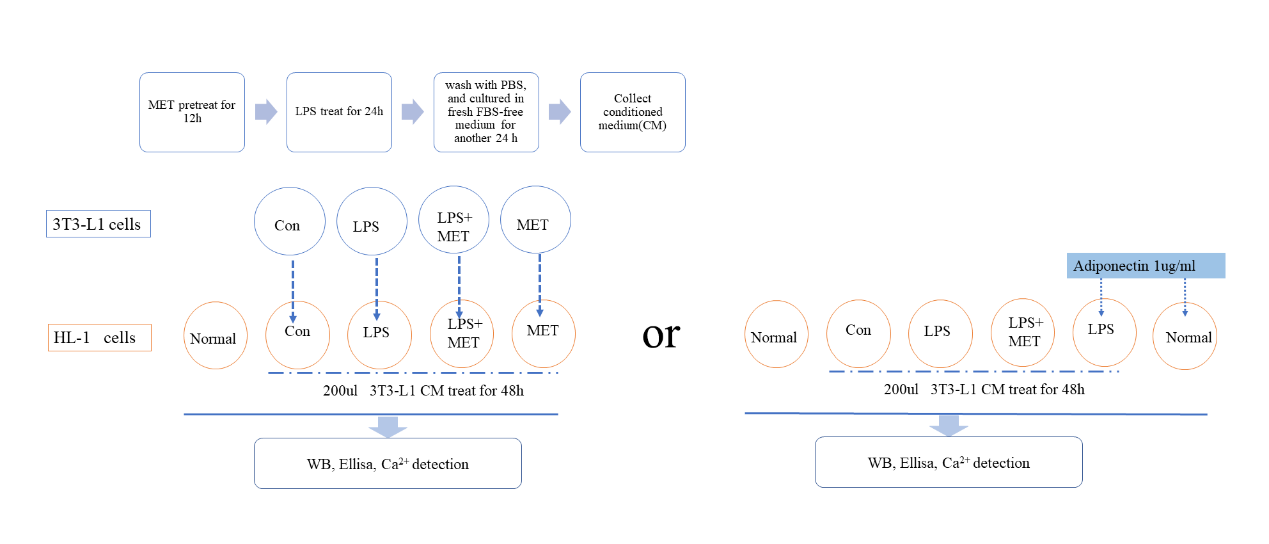


Schematic diagram of the *in vitro* experiment. 3T3-L1 cells were pre-treated with MET, then treated with LPS or treated only with LPS directly. The 3T3-L1 conditioned medium (CM) was then collected for HL-1 cells treatment.

**Supplementary Figure 2.**

**
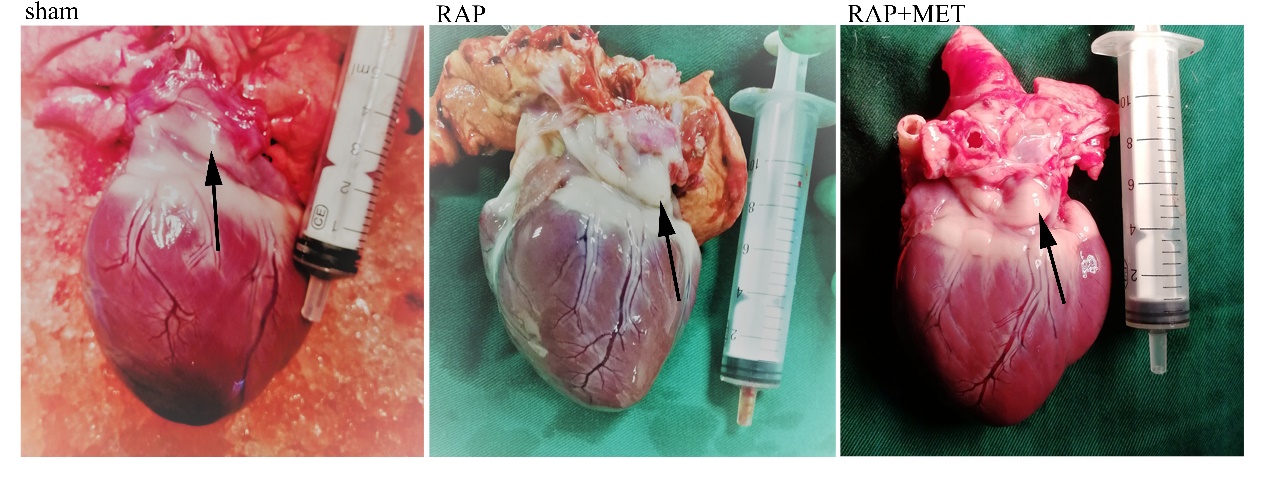
**

Representative posteroanterior view of a gross canine heart specimen. The arrow points to the epicardial adipose tissue (EAT) adjacent to the posterior left atrial wall. The EAT volume of the RAP group was significantly larger than that of the sham group. The EAT volume of the RAP+MET group was smaller than that of the RAP group, but larger than that of the sham group.

**Supplementary Figure 3.**

**
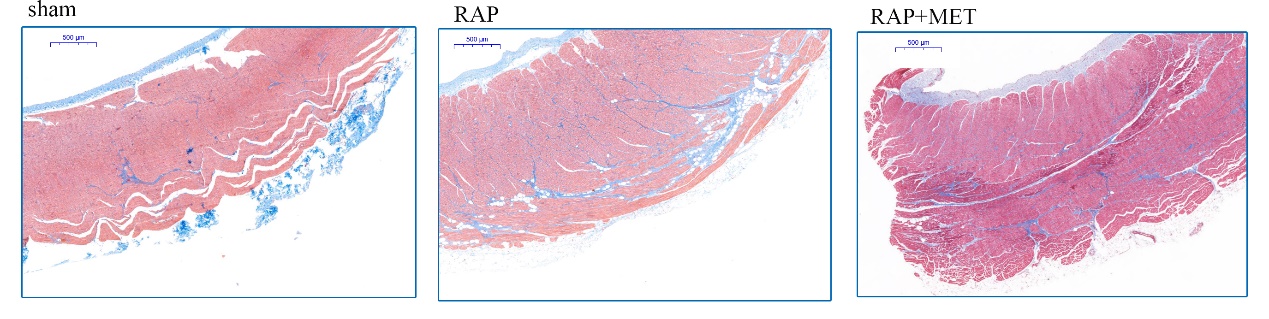
**

Representative HE-stained sections (2.5**×**) demonstrating fatty infiltration of the atrial muscle by epicardial adipose tissue in in the sham-operated group, RAP group, and RAP+MET group. Moderate to severe epicardial adipose tissue infiltration could be observed in the LA posterior wall at a significantly higher level in the RAP group than in the sham operated group and RAP+MET group.

**Supplementary Figure 4.**

**
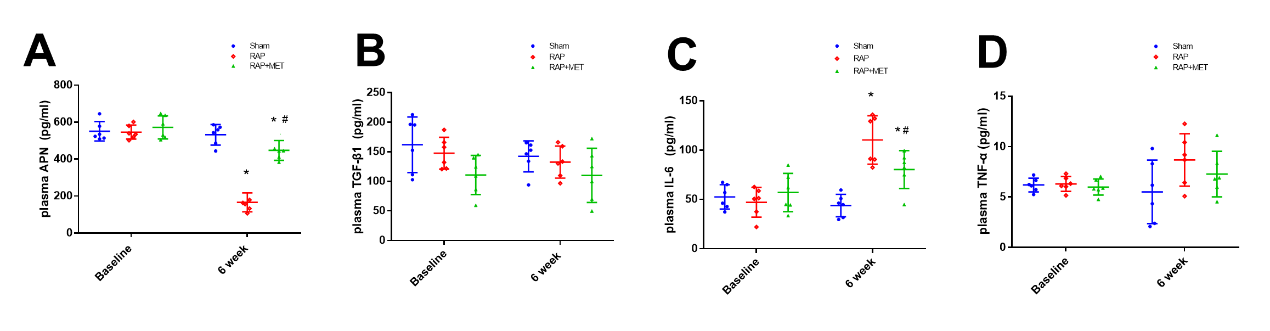
**

Changes in TGF-β1, IL-6, TNF-α and APN plasma concentrations (**A, B, C and D**) at baseline and 6 weeks after surgery in the sham-operated group, RAP group and RAP+MET group (n=6 animals/group). ^*^P <0.05 compared with the sham-operated group; ^#^P <0.05 compared with the RAP group; LA, left atrium; EAT, epicardial adipose tissue; TGF-β1, transforming growth factor-β1; IL-6, interleukin-6; TNF-α, tumor necrosis factor-α; APN, adiponectin.

**Supplementary Figure 5.**

**
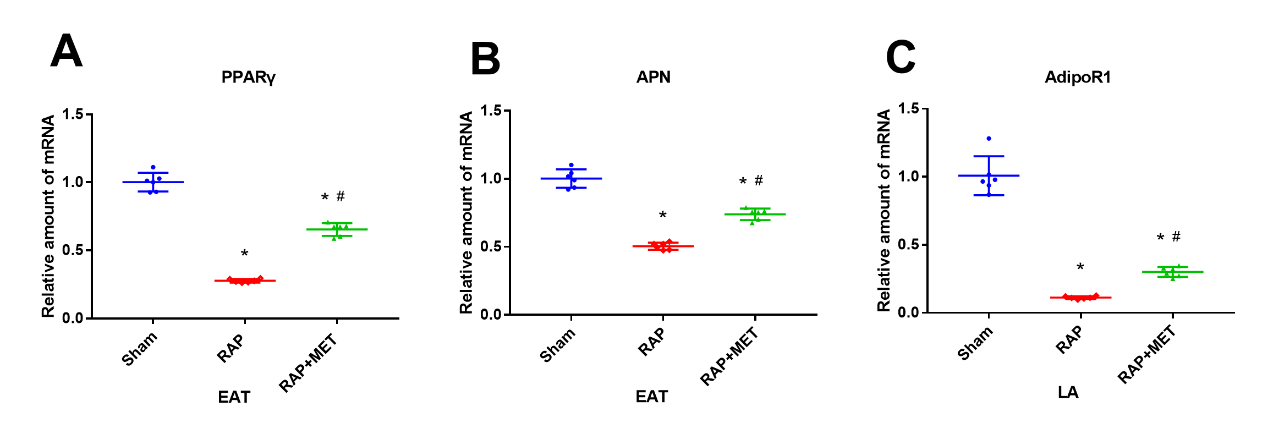
**

mRNA expression of APN and PPARγ in the EAT and AdipoR1 in the LA of the sham-operated group, RAP group and RAP+MET group. (n = 6 animals/group).  ^*^P <0.05 compared with the sham-operated group; ^#^P <0.05 compared with the RAP group; LA, left atrium; EAT, epicardial adipose tissue; APN, adiponectin; PPARγ, peroxisome proliferator-activated receptor gamma.

**Supplementary Figure 6.**

**
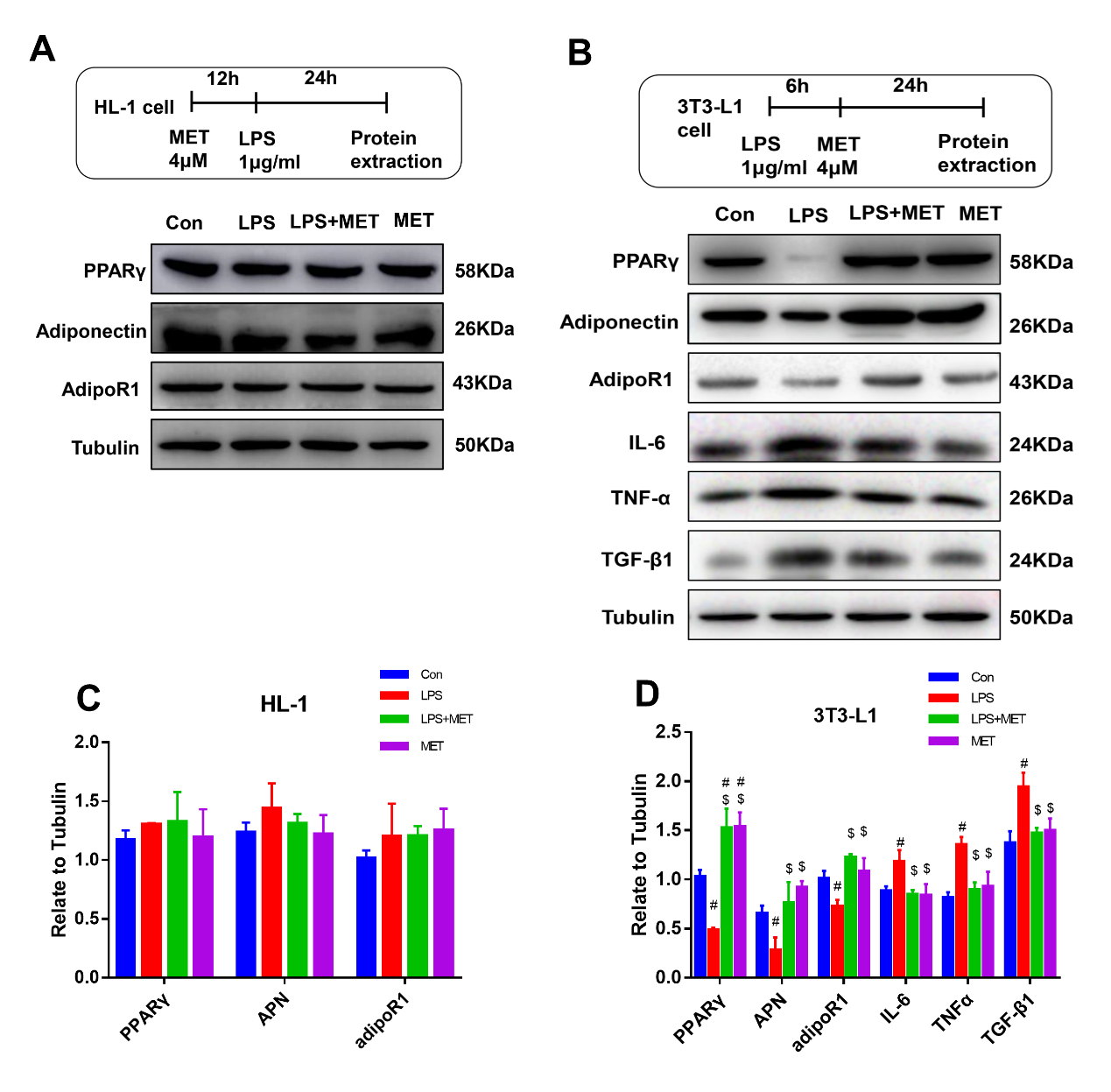
**

(**A, C**) The effect of MET on APN-related protein expression in LPS-treated HL-1 cells. (**B, D**) The effect of MET on inflammatory factors and APN-related protein expression in LPS- pretreated 3T3-L1 adipocytes. All experiments have been repeated 3 times (n=3). ^#^P <0.05 compared with the control group, ^$^P <0.05, compared with the LPS group.
